# Full-Spectrum Neuronal Diversity and Stereotypy through Whole Brain Morphometry

**DOI:** 10.21203/rs.3.rs-3146034/v1

**Published:** 2023-07-25

**Authors:** Yufeng Liu, Shengdian Jiang, Yingxin Li, Sujun Zhao, Zhixi Yun, Zuo-Han Zhao, Lingli Zhang, Gaoyu Wang, Xin Chen, Linus Manubens-Gil, Yuning Hang, Marta Garcia-Forn, Wei Wang, Silvia De Rubeis, Zhuhao Wu, Pavel Osten, Hui Gong, Michael Hawrylycz, Partha Mitra, Hongwei Dong, Qingming Luo, Giorgio A. Ascoli, Hongkui Zeng, Lijuan Liu, Hanchuan Peng

**Affiliations:** 1 SEU-ALLEN Joint Center, Institute for Brain and Intelligence, Southeast University, Nanjing, China; 2 Seaver Autism Center for Research and Treatment, Icahn School of Medicine at Mount Sinai, New York, NY, USA; 3 Department of Psychiatry, Icahn School of Medicine at Mount Sinai, New York, NY, USA; 4 The Mindich Child Health and Development Institute, Icahn School of Medicine at Mount Sinai, New York, NY, USA; 5 Friedman Brain Institute, Icahn School of Medicine at Mount Sinai, New York, NY, USA; 6 Alper Center for Neural Development and Regeneration, Icahn School of Medicine at Mount Sinai, New York, NY 10029, USA; 7 Appel Alzheimer’s Disease Research Institute, Feil Family Brain and Mind Research Institute, Weill Cornell Medicine, New York, NY 10021, USA; 8 Department of Cell, Developmental & Regenerative Biology, Icahn School of Medicine at Mount Sinai, New York, NY, USA; 9 Department of Neuroscience, Icahn School of Medicine at Mount Sinai, New York, NY, USA; 10 Cold Spring Harbor Laboratory, Cold Spring Harbor, NY, USA; 11 HUST-Suzhou Institute for Brainsmatics, JITRI, Suzhou, China; 12 Allen Institute for Brain Science, Seattle, WA, USA; 13 Center for Integrative Connectomics, Department of Neurobiology, David Geffen School of Medicine at UCLA, Los Angeles, CA, USA; 14 State Key Laboratory of Digital Medical Engineering, School of Biomedical Engineering, Hainan University, Haikou, China; 15 Key Laboratory of Biomedical Engineering of Hainan Province, One Health Institute, Hainan University, Haikou, China; 16 Volgenau School of Engineering, George Mason University, Fairfax, VA, USA

## Abstract

We conducted a large-scale study of whole-brain morphometry, analyzing 3.7 peta-voxels of mouse brain images at the single-cell resolution, producing one of the largest multi-morphometry databases of mammalian brains to date. We spatially registered 205 mouse brains and associated data from six Brain Initiative Cell Census Network (BICCN) data sources covering three major imaging modalities from five collaborative projects to the Allen Common Coordinate Framework (CCF) atlas, annotated 3D locations of cell bodies of 227,581 neurons, modeled 15,441 dendritic microenvironments, characterized the full morphology of 1,891 neurons along with their axonal motifs, and detected 2.58 million putative synaptic boutons. Our analysis covers six levels of information related to neuronal populations, dendritic microenvironments, single-cell full morphology, sub-neuronal dendritic and axonal arborization, axonal boutons, and structural motifs, along with a quantitative characterization of the diversity and stereotypy of patterns at each level. We identified 16 modules consisting of highly intercorrelated brain regions in 13 functional brain areas corresponding to 314 anatomical regions in CCF. Our analysis revealed the dendritic microenvironment as a powerful method for delineating brain regions of cell types and potential subtypes. We also found that full neuronal morphologies can be categorized into four distinct classes based on spatially tuned morphological features, with substantial cross-areal diversity in apical dendrites, basal dendrites, and axonal arbors, along with quantified stereotypy within cortical, thalamic and striatal regions. The lamination of somas was found to be more effective in differentiating neuron arbors within the cortex. Further analysis of diverging and converging projections of individual neurons in 25 regions throughout the brain reveals branching preferences in the brain-wide and local distributions of axonal boutons. Overall, our study provides a comprehensive description of key anatomical structures of neurons and their types, covering a wide range of scales and features, and contributes to our understanding of neuronal diversity and its function in the mammalian brain.

## Introduction

Neurons are the fundamental units of nervous systems, and their morphological analysis is crucial to understand neural circuits (Luo, 2021). One salient feature of mammalian neurons is their extensive, long-range axonal projections across brain regions (Zeng & Sanes, 2017). However, our understanding of neuronal morphology and function is limited by the incomplete digital representation of neuron patterns (Peng et al., 2015; Manubens-Gil et al., 2023). Recent studies have focused on more complete representations of neuronal morphology, including both dendrites and axons, using genetic and viral techniques that label neurons sparsely (Ghosh et al., 2011; Kuramoto et al., 2009; Lin et al., 2018; Luo et al., 2016). To produce these representations, multiple imaging modalities, such as serial two-photon tomography (STPT) (Ragan et al., 2012), light-sheet fluorescence microscopy (LSFM) (Keller & Ahrens, 2015; Silvestri et al., 2013) and fluorescence micro-optical sectioning tomography (fMOST) (Gong et al., 2016; Zhong et al., 2021), have been employed. These neuron-labeling and imaging techniques have produced a vast amount of imaging data, primarily hosted by the Brain Research through Advancing Innovative Neurotechnologies (BRAIN) Initiative - Cell Census Network (BICCN) community (BICCN Data Ecosystem Collaboration et al., 2023).

Recent studies emphasize the importance and advances of generating complete neuron morphology reconstructions, particularly long projecting axons (Winnubst et al., 2019; Peng et al., 2021; Gao et al., 2022). However, analyses of the complex arborization patterns of axons in mammalian brains are still limited. Analysis of the dendritic arborization has also been limited to traditionally defined morphological features, but is largely missing the overlay with brain anatomy to yield rich spatial information. Additionally, there has been little work on integrating information from neuronal populations, individual neurons, and sub-neuronal structures at both neuronal arbor and synapse levels (Parekh & Ascoli, 2015). The analysis of large-scale structural data of neurons across various anatomical scales, from whole brain to synapses, remains insufficiently explored.

In our effort to analyze neuronal patterns at different scales, we consider the statistical distributions which quantify both the diversity and stereotypy of neuronal patterns (Peng et al., 2011, 2021). Across different “types” or “classes” of neuronal patterns, a *diversity* metric describes the variety among different types of neuronal patterns and their respective degrees, while a *stereotypy* metric quantifies the level of conservation of patterns within each type. Neurons may differ greatly in their morphological, physiological and molecular attributes (Compston, 2001; The Petilla Interneuron Nomenclature Group (PING), 2008; Zeng & Sanes, 2017; Miller et al., 2020). Despite previous efforts to study the diversity and stereotype of various neuron types, such as hippocampal interneurons (Booker & Vida, 2018), striatal neurons (Surmeier et al., 2011), and cortical neurons (Peng et al., 2021), a systematic analysis at a whole brain level and across multiple scales is yet to be developed.

Our study makes an initial effort in describing the diversity of conserved morphological patterns of neurons at various anatomical and spatial scales in the context of whole mouse brains. Using a massive number of light-microscopic images of mouse brains generated by the community of Brain Research through Advancing Innovative Neurotechnologies (*BRAIN)* Initiative - Cell Census Network (BICCN), we performed an analysis of 3.7 peta-voxels of images with which we also reconstructed thousands of annotated neurons, and developed one of the largest available multi-morphometry datasets. By analyzing patterns of neurons at six continuous structural scales, we discovered conserved morphological modules and motifs distributed throughout entire brain. This effort allows us to develop both a comprehensive picture of the whole brain anatomy, as well as a detailed, multi-scale description of neuron morphologies. Furthermore, we also attempted to establish a model explaining how features of different scales have complementary effects on morphological characterization. By combining the diversity and stereotypy scores at different scales, we visualized and quantified various anatomical modules of a brain, which were grouped together using morphology, projection, and lamination information, at single-neuron resolution.

## Results

### Brain mapping of multi-morphometry data generated from peta-voxels of neuron images

**Figure 1.**
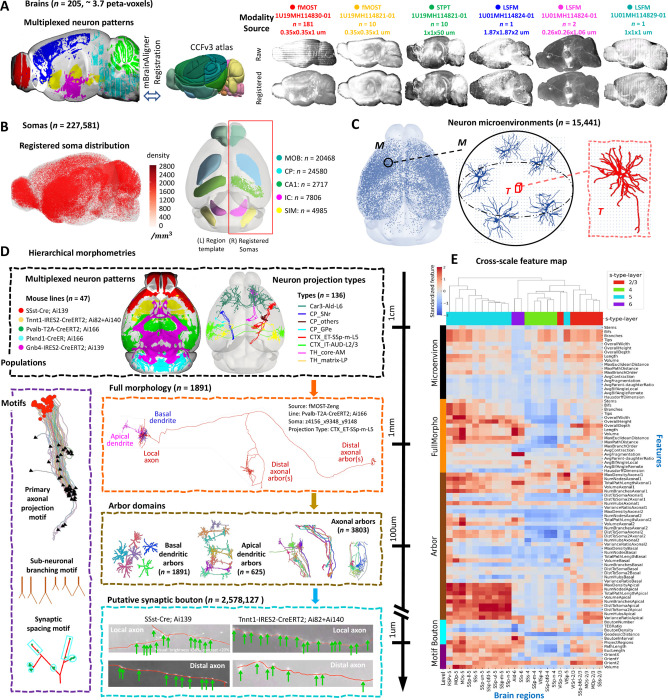
Multiscale morphometry analysis from multi-modal mouse brain images. **A.** The multi-modal mouse brain dataset IMG205 comprises 205 brains (3.7 peta-voxels) of 3 different modalities (fMOST, STPT, and LSFM) obtained from 4 BICCN projects (grant identifiers: 1U19MH114830-01, 1U19MH114821-01, 1U01MH114824-01, 1U01MH114829-01) and one collaboration project involving Southeast University, Allen Institute, and other organizations. Left: A multiplexing view displays salient voxels on the sagittal middle sections of 6 mouse brains from different sources. The salient voxels are colored by image sources. Middle: The CCFv3 atlas that all brains are registered to, using the cross-modal registration tool mBrainAligner. Right: Representative sagittal maximum intensity projections of whole-brain images from each modality and source. Imaging modality, research group, the number of brains collected, and typical voxel size are specified at the top. The brain images are diverse in labeling and resolution but comparable after mapping to the CCFv3 space. We provide comprehensive metadata for all brains in Supplementary Table S1. **B.** Left: Sagittal view of the spatial distribution of 227,581 semi-automatically annotated somas on the CCFv3 template, along with their densities (color bar). Each soma is represented by an individual dot. Right: Horizontal projection of five regions (color-coded) along the anterior-posterior (AP) axis (left) and respective soma locations as dots (right). **C.** Left: Horizontal projection of auto-traced dendritic morphologies (SEU-D15K). Middle: Dendritic microenvironment (M) representation for each neuron (target). A microenvironment is a spatially tuned average (see Methods) of the most topologically similar neurons (up to six neurons, including the target neuron) within a distance of 249 μm from the target neuron. Right: Morphology of the target neuron within the microenvironment on the left. **D.** Multiscale morphometry. Hierarchical representation including representative visualizations for six scales of morphometrics ranging from centimeters to micrometers, i.e., neuron population (mouse lines and projection types), full morphology, arbor, motif, synaptic site, and the microenvironment displayed in panel C. **E.** Heatmap of the cross-scale feature map for lamination subtypes of cortical neurons (s-type-layer). To calculate the full feature set of each neuron (each row is one morphometric feature), we combined features from multiple morphometry scales (colored grouping in the left vertical axis), and the regional features were estimated by the mean feature vector of all neurons in that region. The lamination subtypes are hierarchically clustered and ordered based on the dendrogram (colored grouping in the top horizontal axis). Denomination of all regions and soma types (s-types) is based on the CCFv3 atlas. Soma types (s-types) with their soma located in the same cortical lamination are grouped together.

We assembled one of the largest collections of single-neuron morphology data in mice through five joint projects involving BICCN and partners. This 3.7 peta-voxels dataset included 205 whole-brain images the micrometer and sub-micrometer resolutions imaged using fMOST, STPT, and LSFM, respectively (Figure 1A and Supplementary Table S1). We call this image dataset IMG205 to simplify the subsequent description. We analyzed these multi-modal images to investigate the modular organization of brains and associated patterns across anatomical scales. To facilitate an objective comparison of neuronal patterns across different imaging modalities and conditions, we registered all IMG205 images to the Allen Common Coordinate Framework (CCF) version 3 (CCFv3) (Wang et al., 2020) atlas, using a cross-modality registration tool mBrainAligner (Li et al., 2022; Qu et al., 2022) (Figure 1A, Methods). Although IMG205 contains primarily fMOST images (191/205), the inclusion of other imaging sources provides a valuable generalizable framework for future applications to additional modalities. Indeed, the sparsely labeled neuron populations in different brains could be accurately aligned to study the colocalization relationship of their patterns (Figure 1A).

To demonstrate the utility of our data analysis framework, we produced quantitative descriptors of patterns at various morphological scales, from whole brain to the synapse resolution. To do so, we developed a cloud-based Collaborative Augmented Reconstruction (CAR) platform (Peng et al., 2023, unpublished) as the software platform including several computational tools to generate high-throughput multi-morphometry with high precision. We performed semi-automatic annotation of a total of 227,581 neuronal somas from 116 fMOST brains (Figure 1B; Supplementary Table S1; Supplementary Table S2) using an initial automatic soma detection, followed by collaborative annotation through a mobile application called Hi5 available through the CAR platform. We call this soma dataset SEU-S227K, including detailed information of brain ID, soma-location in 3-D, and registered brain region (Supplementary Table S3). As neurons were often labeled with different degrees of sparsity in these brains, we captured the large variation of soma distribution in various brain samples by annotating both brains with very sparsely labeled neurons and also brains with densely labeled neurons. Overall, in 78% (91/116) of the brains in SEU-S227K, there are more than 100 annotated somas. Spatially, among 314 non-fiber-tract regions in CCFv3 (CCF-R314, Methods), 295 regions have annotated soma (Figure 1B). We also examined the variation of soma density in specific brain regions, for instance, while each of the 132 regions has more than 100 annotated somas, caudoputamen (CP) and the main olfactory bulb (MOB) have >20,000 somas and high densities of up to 1712 and 2578 somas/mm^3^ respectively.

We then traced both the dendritic and axonal morphologies of individual neurons whose somas had been annotated. For dendrites, we constructed a database, called SEU-D15K, which contains 15,441 automatically reconstructed dendritic morphologies in 3-D. We cross-validated the brain-wide reconstructions in SEU-D15K with the dendrites of 1891 manually curated neurons and found very similar dendritic distributions of bifurcation and surface areas (Supplementary Figure S1A). Overall, SEU-D15K dendrites share a similar appearance in terms of their morphological features, although sometimes dendrites with somas in proximity, i.e., those in the same brain regions, may cluster closely in the dendrogram (Supplementary Figure S1B). To derive a spatially tuned dendritic feature vector with high discrimination power, here we extended our recent spatial tensor analysis of dendrites for human neurons (Han et al., 2023) to analyze these mouse dendrites in SEU-D15K, and developed a *dendritic microenvironment* representation to characterize the local neighborhood information around a target dendrite (Figure 1C; Methods). Because in mouse brains we have more precise location information of neurons than in human surgical tissues (Han et al., 2023), the dendritic microenvironment can be intuitively constructed to describe the spatially tuned dendrite structures (Methods). In this way, we produced 15,441 dendritic microenvironments corresponding to SEU-D15K and used this approach to quantify the dendritic diversity and stereotypy as shown later.

Using our framework of multiscale morphometry (Figure 1D; Methods) that spans resolution levels from centimeter to micrometer, we analyzed the multiplexed neuronal patterns (Figure 1A, Figure 1D) and dendritic microenvironments (Figure 1C), as well as the fully reconstructed neuron morphologies. Here we constructed a dataset SEU-A1891 that contains fully traced 3-D morphologies of 1891 neurons, including their complete dendrites, proximal axonal arbors, and distal axon arbors (Figure 1D) (Peng et al., 2021). We specifically extracted 3,803 densely branching axonal arbors, 2,516 dendritic arbors (1,891 basal and 625 apical), as well as the primary projection tracts connecting such arbors (Figure 1D). Then we identified the diversified patterns, each sufficiently conserved as a “motif”, identifying a number of axonal bundle motifs. In addition, we detected 2.58 million axonal varicosities from the axonal arbors to model putative synaptic sites, and accordingly pinpointed the respective synaptic motifs (Figure 1D).

Our analytics framework covers six major scales of neuronal patterns (Figure 1D): Neuronal populations, dendritic microenvironments, single-cell full morphology, sub-neuronal dendritic and axonal arborization, structural motifs, axonal boutons, along with quantitative characterizations of the diversity and stereotypy of patterns at each level as reported hereafter. We defined and extracted a number of features, all standardized using brain mapping to the CCFv3 atlas, to characterize properties of brain regions as well as individual neurons whenever possible (Figure 1E). Cross-scale feature maps demonstrate high potential for cell typing and subtyping, with anatomically similar regions generally exhibiting analogous morphology throughout the whole brain (Supplementary Figure S2A). Moreover, lamination and projection patterns emerge as prominent factors in grouping subtypes of cortical neurons, based on cross-scale features (Figure 1E, Supplementary Figure S2). Our analyses also found that broadly distributed yet highly discriminating features across multiple scales could be integrated (Supplementary Figure S2).

### Inferring brain modules using multiplexed brains

For neuronal patterns visible in the range of millimeters to centimeters, we analyzed the diversity and stereotypy of neuron populations labeled in IMG205 (Figure 1D). Quantifying the conservation or reproducibility of neuronal patterns (stereotypy), in functionally established anatomical regions helps define whether these patterns are sufficiently consistent to make biological inferences. On the other hand, capturing the diversity of these patterns not only confirms anticipated differences across brain regions, but also validates the accuracy in aligning multimodal images during brain multiplexing.

**Figure 2.**
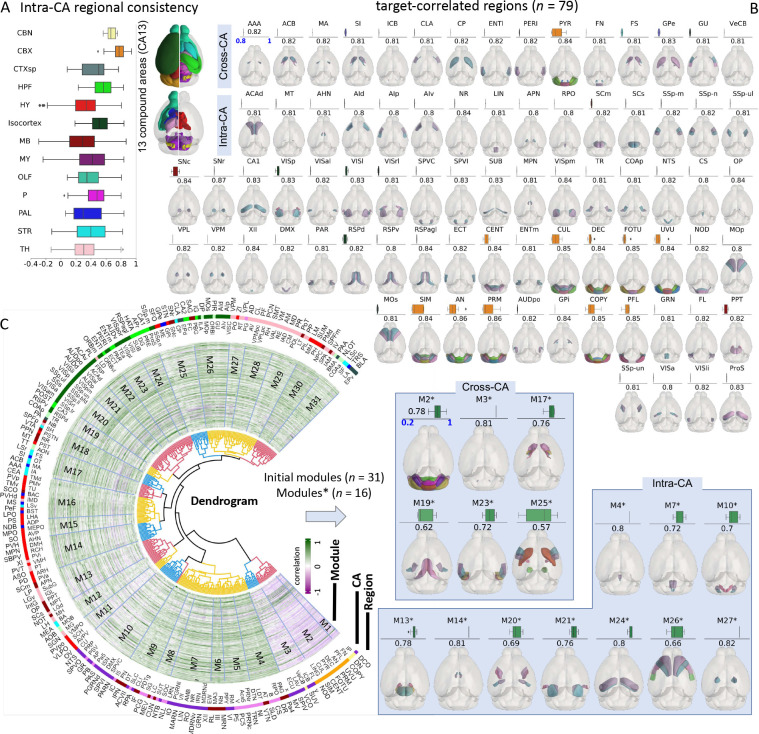
Modules inferred from multiplexed brains. **A.** Intra-Compound Area (intra-CA) consistency in neurite density among the analyzed brain images. The consistency is the correlation between all pairs of regions within a compound area, defined as the Spearman coefficient of brain-wide neurite densities for each pair of regions. Left: Box plot of the intra-CA consistencies for 13 compound areas in the brain (color-coded). Right: The 13 compound areas projected on a horizontal view of the CCFv3 template. A compound area is a super-region composed of a subset of anatomically and functionally correlated CCFv3 regions (CBN: cerebellar nuclei, CBX: cerebellar cortex, CTXsp: cortical subplate, HPF: hippocampal formation, HY: hypothalamus, isocortex, MB: midbrain, MY: medulla, OLF: olfactory areas, P: pons, PAL: pallidum, STR: striatum, TH: thalamus). **B**. Horizontal projections on the CCFv3 template of regions with a Spearman correlation coefficient of at least 0.8 with the target region (specified at the top of each image). Each image is accompanied by a box plot that shows the distribution of the pairwise correlations between these regions and the target region, with the box colored by CA as in panel A. An intra-CA region set indicates all regions in the current region set are within the same compound area, and cross-CA span across at least 2 compound areas. **C**. Whole-brain co-occurrence modules. Left: Circular heatmap representing the neurite density distribution for each CCFv3 brain region (N=314) as radial 191-element vectors (number of brain images). The dendrogram shows how the brain regions cluster together to form modules. Labels for each region are specified on two outer layers of the graph, the corresponding compound areas are labeled with the colored circle. Right: Cross-CA and intra-CA tightly inter-correlated modules inferred from the dendrogram, with their modular consistencies (pairwise Spearman correlations, as in panel A) shown in the box plots on the top of the brains.

We developed an algorithm to segment neurites in IMG205 (Methods), and used the co-occurrence of these neurites over the entire set of image samples to infer the diversity and stereotypy of the respective neuron populations. We grouped all 314 brain regions defined in CCFv3 into 13 larger regions (combined areas, CAs) of the CCFv3 taxonomy each corresponding to sets of functionally related brain regions (Figure 2A). We found that several CAs, e.g., isocortex, cerebellar cortex (CBX) and cerebellar nuclei (CBN), have more tightly correlated intra-areal neurite patterns than other CAs (Figure 2A). Within each CA, the labeled neuron populations always have a positive correlation (Figure 2A), implying the colocalized brain patterns in IMG205 are generally consistent in general despite the heterogeneity of specimen preparation and imaging.

We sought to identify highly correlated brain regions for each of the 314 CCFv3 regions (“target”), resulting in the discovery of 79 sets of individual regions that exhibit a strong correlation (no less than 0.8) with their target regions. For each of these sets, we identified one or more matching brain regions whose neurite patterns correlate most strongly with the patterns in the target (Figure 2B, Supplementary Table S4). 64 sets involve regions in the same CAs (intra-CA), while the other 15 involve regions from different CAs (cross-CA). All these 79 sets, however, turn out to be immediate neighbors that share region borders (Figure 2B). Such a strong correlation of neuronal patterns in neighboring brain regions suggests neurite signal across each pair, which most likely contains continuous neuron projections through or arborization covering them. Examples include the caudoputamen and globus pallidus - external segment (CP-GPe) pairs for which we reported single neuron level projection in a previous study (Peng et al., 2021). These results suggest that stereotyped “connections” of neurite signals are identifiable in spite of potential imperfect hierarchical groupings of brain regions in an existing brain atlas such as CCFv3.

The observation above motivated us to further search for modules of brain regions that share the co-occurring neurite-signal as tight clusters (Figure 2C). We identified 31 non-overlapping intercorrelated initial modules from the hierarchical dendrogram (Methods). Six initial modules are intra-CA, and 25 are cross-CA (Figure 2C). For most initial modules, the regions identified are neighboring, with exceptions. We determined coherent modules by including only brain regions that appear frequently in the hit list of the target-correlation search (Figure 2B). In this way, we obtained 16 modules (Figure 2C, Supplementary Table S5), which highlight hubs of co-occurring neurite signals. For example, M25* contained 7 regions, i.e., primary somatosensory area - mouth (SSp-m), primary somatosensory area - nose (SSp-n), globus pallidus - external segment (GPe), globus pallidus - internal segment (GPi), substantia nigra - compact part (SNc), substantia nigra - reticular part (SNr), and caudoputamen (CP). This module is consistent with the diagram of basal ganglia circuits (Gerfen & Surmeier, 2011). The module M26* contained 6 regions, namely primary motor area (MOp), secondary motor area (MOs), gustatory areas (GU), agranular insular area - dorsal part (AId), agranular insular area - ventral part (AIv), and agranular insular area - posterior part (AIp). This module is consistent with a previous study, which reported that the regions GU and AId serve as inputs for the upper limb area of MOp (MOp-ul). Moreover, all six of the regions in M26* are identified as outputs of MOp-ul (Muñoz-Castañeda et al., 2021). Overall, our data indicates a highly modularized brain organization, whose parcellation we sought to further examine.

### Discovering brain parcellation using dendritic microenvironments

**Figure 3.**
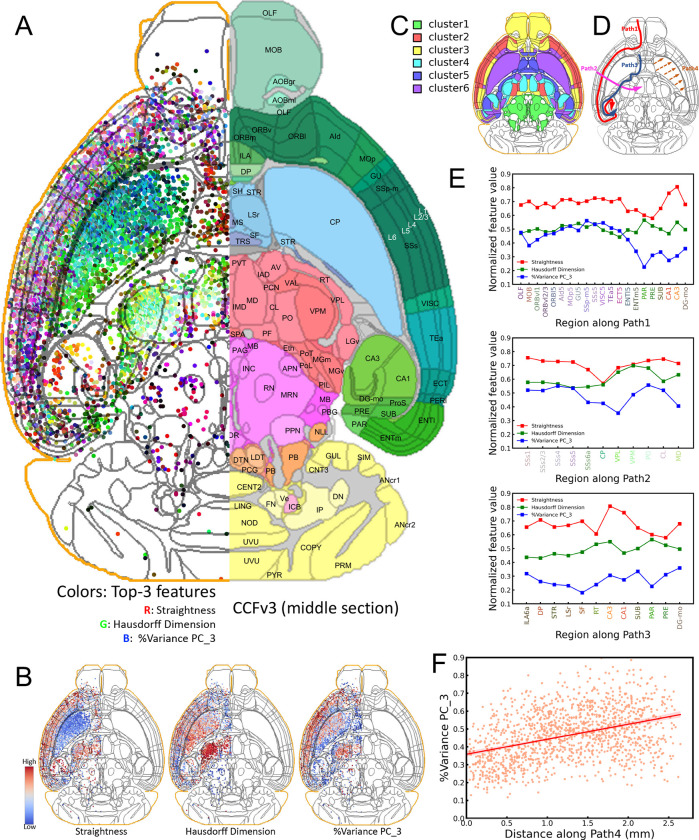
Feature distributions of dendritic microenvironments across the whole brain. **A.** Left: the top discriminating three features of microenvironments projected on the middle axial section of the CCFv3 atlas represented as colored points. Those were selected using the minimum Redundancy – Maximum Relevance (mRMR) algorithm from a set of 24 morphological features and were then normalized and histogram-equalized to unsigned 8-bit integer space to enhance visualization. Right hemispheric microenvironments were flipped to the left hemisphere. The three features are average straightness, Hausdorff dimension, and variance percentage of PC_3, representing fiber bending, fractal complexity, and spatial uniformity, respectively. Notably, only microenvironments within a 1 millimeter range of the middle axial section were included for clarity. The outer boundary of the CCFv3 template is indicated by the orange outline on the left. On the right, the CCFv3 atlas is depicted. The color scheme follows the convention of the CCFv3 atlas, with the green-blue color system representing cortical regions, cyan colors indicating striatal regions, red colors indicating thalamic regions, pink colors representing midbrain, and yellow colors representing cerebellar regions. **B**. The distribution of each of the top-three features separately, following the same feature standardization as in panel A. **C**. Middle axial section colorized by clusters predicted based on the mean and variance of the top-three features of all microenvironments in the region. K-Means clustering was used for classification. **D**. Schematic representation of four paths along which we measured the feature distribution, including intra-area cross-region (Path1, along cortical regions), cross-brain area (Path2, from cortex to striatum and thalamus and Path3, from cortex to striatum, thalamus and midbrain), and intra-region (Path4, CP region). **E**. The distribution of regional mean features along Path1, Path2, and Path3. We colored the lines following the corresponding color scheme for each channel displayed in Panel A. Additionally, the median feature value of the region colors the region name. **F**. The gradual spatial change in the variance percentage along the radial direction of Path4, specifically showing the CP region.

We used the diversity and stereotypy of single neuron morphological patterns to further delineate brain modules. We first examined the dendritic patterns of individual neurons. For SEU-D15K (Figure 1C), the local dendrites are distributed in the majority of CCFv3 regions (222/314). To characterize neuronal architecture in local neighborhoods, we extracted a 24-dimensional feature vector for each dendritic microenvironment to aggregate both the dendritic morphology of individual neurons and the spatial relationship of neurons in a small neighborhood (Methods). Next, we mapped the top three discriminating features selected using a minimum-Redundancy-Maximum-Relevance (mRMR) algorithm (Peng et al., 2005) to the CCFv3 atlas to produce a 3-D brain-wide RGB-coded microenvironment map, with each channel corresponding to one feature (Figure 3A). Alternatively, users may select their preferred feature channels or merge them to reduce dimensions, enabling them to visualize and analyze the data more effectively.

Whether dendritic features can be leveraged to distinguish cell types is debated (DeFelipe et al., 2013; Polavaram et al., 2014), but without complete and accurate dendrite reconstructions we are clearly limited in these efforts. Unfortunately, with the current labeling techniques it is still challenging to reconstruct without errors the entire dendrite arborization of a neuron. For pyramidal neurons, often it is difficult to reconstruct precisely both basal and apical parts of dendrites, as apical dendrites can also extend substantially. Neuron partition methods such as G-Cut (Li et al., 2019) cannot avoid loss of information, either. In our dendritic microenvironment approach, we mitigated these problems by prioritizing accuracy over completeness. We considered only precisely reconstructed local dendrites surrounding somas to improve classification.

One remarkable observation is that despite the limitations of the approach, the microenvironment map shows clear boundaries that align with the primary CCFv3 region borders (Figure 3A). For example, CP neurons are clearly distinct from cortical neurons. Cortical layers can also be discriminated based on these features, adding on observations from conventional soma-density method (Kim et al., 2017; Wang et al., 2020), axon projections (Peng et al., 2021), or a full description of the apical-basal dendrites of cortical neurons. Indeed, while each of the three color-coding features has a different distribution (Figure 3B), they jointly define a number of anatomical details that are consistent with CCF.

Based on the diversity of brain regions as indicated by the dendritic microenvironments, we generated 6 major clusters of regions (Figure 3C). In the shown example, most laminated cortical neurons share similar feature patterns and thus are grouped together in one of the major clusters, although they could be further clustered hierarchically. Hippocampal neurons in CA1 and CA3 are clustered away from cortical, striatal, and thalamic neurons (Figure 3C). Indeed, the hippocampal neurons have similar average straightness and Hausdorff dimensions like most other cortical neurons but differ in variance percentages (Figure 3B). CP neurons, however, have a distinct pattern compared to other striatal neurons (Figure 3A, Figure 3C).

Within each microenvironment cluster, however, neurons show evident stereotypy. To measure the conservation and transition of these features within or across brain regions, we took an approach guided by the definition of four axial projection paths (Figure 3D). The first path follows the tangential flow along the lamination of cortical layers. Cortical neurons share relatively stable features until entering the entorhinal area, lateral part, layer 5 (ENTl5) (Figure 3E - Path1). The second path, orthogonal to the first one, clearly reveals the valleys of two features when entering and leaving CP (Figure 3E - Path2). The third path following one side of the border of CP and nearby regions shows the different distributions for the three features, which means that local dendrites along this path have strong heterogeneity. Thus, along the third path, there is a high likelihood that a variety of cell types can be encountered (Figure 3E - Path 3). The fourth path demonstrates a smooth, and indeed almost linear, gradient of the microenvironment features for CP (Figure 3F). We did not discover this gradient using alternative approaches, even with fully reconstructed axons as in a previous study (Peng et al., 2021).

Based on dendritic microenvironments, one may perform an exhaustive survey of many paths across different 3-D anatomical areas. Interesting examples include but are not limited to the stereotypy discovered in analyzing the middle sagittal and coronal sections (Supplementary Figure S4), and the left-right symmetry of feature patterns in two hemispheres of the brain (Supplementary Figure S4, Figure S5). Overall, the microenvironment analysis is consistent with established brain parcellation in CCFv3 while offering finer detail with respect to the dendritic characteristic within each brain region.

### Detecting primary distributions and key morphological variables of fully reconstructed neurons

We next analyzed the fully reconstructed neurons with complete axons and dendrites in SEU-A1891. While the neuron reconstructions were manually edited by multiple annotators to ensure the correctness of branching patterns, the limited precision of spatial (3-D) pinpointing in manual annotation caused the skeleton of almost every neuron to deviate slightly from the center of the image signal of the skeleton. Therefore, we developed an automatic approach to correct such aberration (Methods) (Li et al., 2023), and generated precisely centered neuron skeletons. This development was also leveraged for the subsequent analyses of axonal varicosities.

**Figure. 4:**
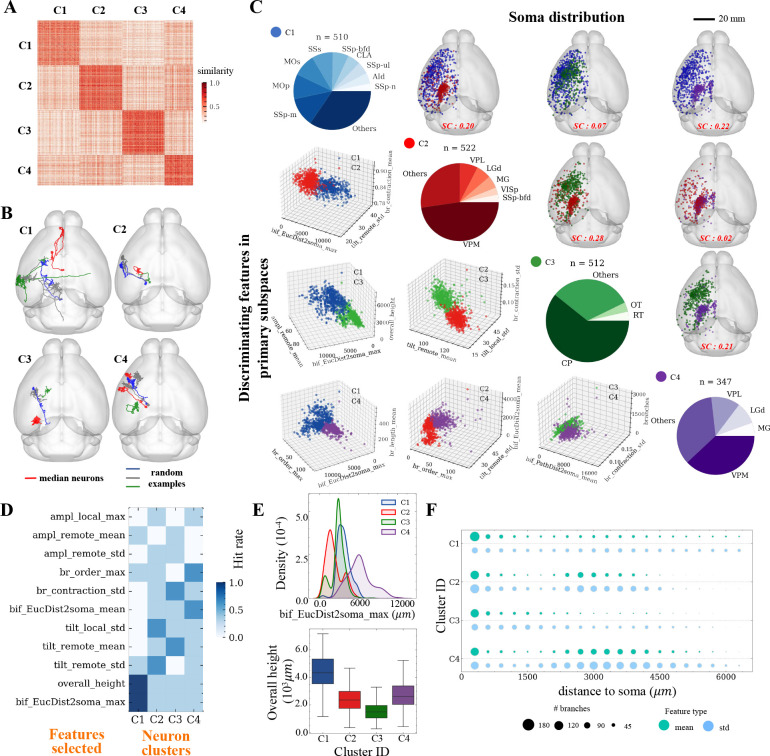
Anatomical characterization of whole-brain fully reconstructed neurons. **A.** Heatmap of pairwise neuron similarities. Each row and column is one neuron. The color shows similarity values between pairs of neurons calculated as the product of the cosine distance between standardized morphological features of each neuron over the exponential of normalized between-soma distance. Neurons were categorized into 4 clusters: C1, C2, C3, and C4 using Spectral clustering (see Methods). **B.** Horizontal projections on the CCFv3 template of representative neurons of each cluster, including a median feature neuron and three randomly selected neurons. **C.** A pair-plot displays the composition of neuron types within each cluster (pie plots in the main diagonal), as well as soma spatial distributions of cluster pairs (upper triangle) and 3D scatter plots showing pairwise separability of neurons from each cluster (color-coded) with respect to the top 3 discriminating features between cluster pairs (lower triangle). To assess the difference in soma distribution, we used the average Silhouette Coefficient (SC), specified in red in the upper triangle. The top 3 discriminating features in the lower triangle were selected through mRMR. The viewpoints of the 3D scatter plots (lower triangle) were adjusted to optimize the visualization of cluster separability. **D.** Heatmap of the number of times (hit rate) a feature was selected by mRMR as a top discriminating feature of the clusters. We selected the features using the mRMR algorithm in 6 independent rounds, where each round corresponded to a separate cluster pair, and we recorded the top 3 features. The hit rate for each cluster is the frequency of the feature being selected in the top 3. **E**. Top: Density plot of maximal Euclidean bifurcation-to-soma distance of all bifurcations for all neurons in each cluster. Bottom: Boxplot of overall heights (maximal span along y-axis) of neurons between clusters. **F**. Matrix visualization of the mean (light green) and standard deviation (std; light blue) of the branch numbers (represented as dot size) with respect to the bifurcation-to-soma distance. Each row is one cluster, and each column is the distance at which we measured branch numbers (30μm intervals).

The entire set of SEU-A1891 neurons is brain-wide distributed, projecting to and covering most major brain regions. These neurons extend dozens of millimeters (Figure 4). It has been often observed that different neuron classes are poorly discriminated by global morphology where features such as length and branching number are considered (Liu & Qian, 2022; Peng et al., 2021). To overcome this limitation, we registered the whole set to CCFv3 using mBrainAligner. The standardization of these neurons’ coordinates allowed us to use the spatial adjacency of neurons to augment morphological features. Specifically, we generated a similarity matrix of 47 morphological features of the 1,891 neurons, and used the spatial adjacency of neurons as a coefficient matrix to finetune the morphology similarity (Methods, Supplementary Figure S6). In this way, spatially distal neurons are less likely to be clustered together as the result of potentially incorrect matching of morphological features. Indeed, we were able to produce 4 clusters of full neuron morphologies (Figure 4A), even if the locations of their somas did not appear visibly separated in 3-D space (Figure 4B). Visual inspection of examples of neurons in distinct clusters confirms their difference in appearance (Figure 4B). Inspection of the soma-distribution of the neurons in the cluster indicates that C1 consists of cortical neurons; C2 and C4 contain mostly thalamic neurons and a few cortical neurons; and, most C3 neurons are located in the striatum (Figure 4C). However, we also noticed that 9%, 25%, 33%, and 14% of neurons innervate from non-dominant brain areas for clusters C1, C2, C3, and C4, respectively. Interestingly, when each pair of the four clusters was screened, the two clusters being compared appeared to be separable even with only three morphological features selected using the mRMR algorithm, although these characterizing features were different in each case (Figure 4C - lower triangle).

The overall consistency between our *de novo* clustering outcomes and the known primary cell types in the mammalian brain prompted us to dissect the most discriminating neuronal features for each cluster (Figure 4D). We found the most discriminating features vary among clusters (Figure 4D). At whole-brain scale, the most prominent features were the ‘bifurcation distance to the soma’ (‘bif_EucDist2soma’), ‘overall height’, and ‘remote tilt angles.’ It is also clear that one single feature cannot separate these 4 clusters (Figure 4E), but the top features (Figure 4D) can jointly characterize neuron clusters. On average, C1 neurons have a relatively smaller chance to have large distal arbors, while they typically project far away (Figure 4E–F). Bifurcations of C2 neurons normally are close to somas (Figure 4E). C3 neurons rarely have distal arbors, and have a smaller overall height. C4 neurons correlate with C2 spatially and anatomically, and have comparable branching patterns. However, C4 neurons have a substantially greater bifurcation-to-soma distance (Figure 4C, Figure 4E–F). Note that C2 and C4 consist of mostly thalamic neurons, thus the great difference between C2 and C4 indicates there could be two neuron subtypes in these thalamic regions.

### Conserved neuron arborization encodes cortical anatomy

**Figure 5.**
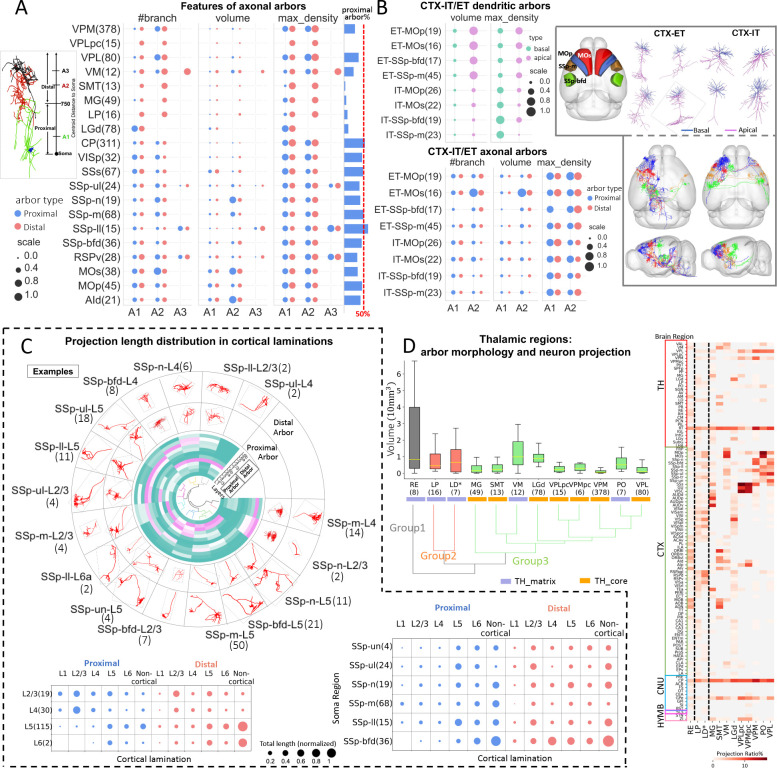
Morphological stereotypy and diversity in neuronal arbors. **A.** Matrix visualization of normalized morphological features (represented as dot size) of axonal arbors for 20 types based on soma location in the CCFv3 (s-types) in which the number of neurons exceeds 10. There is one matrix for each feature of interest (number of branches, volume and maximum density). Each row is one s-type, with the number of neurons specified in parentheses. Each column is a neuronal arbor. The blue and red dots represent the features of proximal and distal axonal arbors respectively, and the ordering of arbors was determined based on their distance-to-soma values. The top left sketch is an exemplar illustration of the categorization of proximal and distal arbors, and their orderings (A1, A2, A3). The arbor types (proximal and distal) were determined by their distance from the max density compartment to somas, where a max density compartment refers to the compartment containing the maximal number of compartments within a 20 μm radius. The histogram on the right displays the average percentage of proximal arbors for each s-type. The parenthetical number after the region name indicates the number of neurons used in that region. **B**. Matrix visualizations of normalized morphological features of dendritic arbors (top left) and axonal arbors (bottom left) of two major cortical neuron projection classes - extratelencephalic (ET) and intraelencephalic (IT) for 4 cortical regions. Dendritic arbors are divided into basal (light green) and apical (light purple), and visualization is analogous to panel A. The top-right component shows horizontal projections of the analyzed regions on the CCFv3 template and representative dendritic morphologies for each region. The bottom-right component shows horizontal and sagittal projections of axonal morphologies for ET (left) and IT (right) neurons mapped in the standard template. **C**. Axonal arbor morphologies and projection distributions of lamination subtypes of cortical SSp neurons, across cortical laminations. Top: Circular heatmap of the projection strengths, measured as normalized total length, across cortical laminations (radial vectors) of 16 SSp subtypes (the number of neurons of each type is specified in parentheses). Two outer layers in the plot show representative examples of proximal and distal axonal arbors. The dendrogram in the center of the plot shows hierarchical clustering based on the projection lengths.. Bottom: Matrix visualization of the projection strength for lamination subtypes (rows on the left) and soma-types (rows on the right). Each column is a cortical layer. **D**. Left: Box plot showing the arbor volume of 12 thalamic neuron types. The dendrogram shows groups obtained by hierarchical agglomerative clustering based on the combination of 8 morphological features (mean and standard deviation of ‘#branch’, ‘volume’, ‘max_density’, ‘dist2soma’) and their projection strength vector across the brain regions. ‘TH_core’ and ‘TH_matrix’ represent the thalamic core and thalamic matrix neurons. Right: Heatmap of the whole-brain projection strength distributions for the 12 types. Each row is a projection region, grouped by their brain areas, which are highlighted at the left of the heatmap. Each row is an s-type region for the analyzed neuron sorted according to the clustering results of the left panel. Given that TH_core only has 3 neurons, only the projection class TH_matrix of LD neurons (LD*) is displayed.

**Figure 6.**
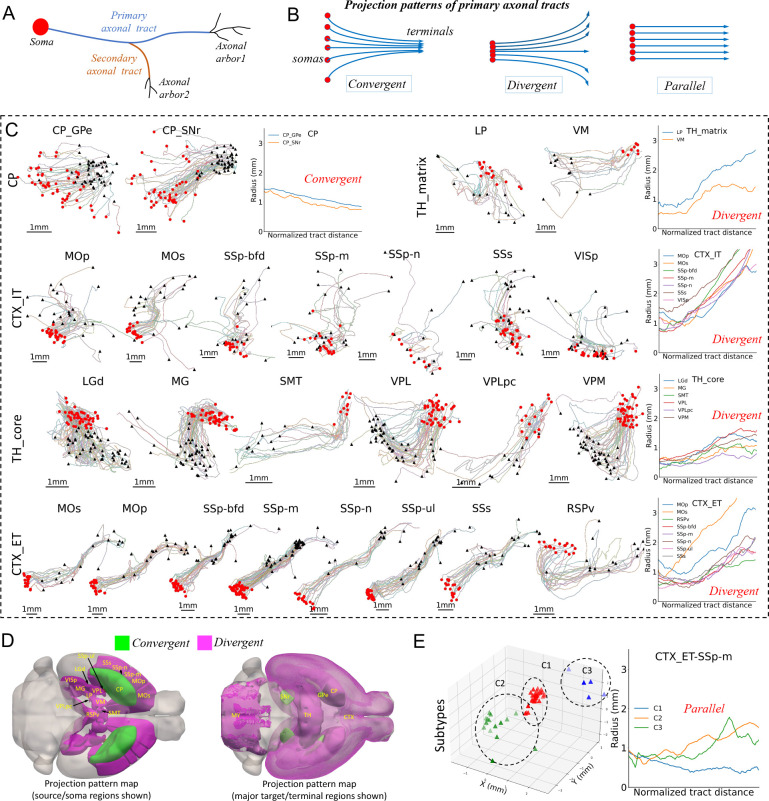
Projection patterns and anatomical insights from primary axonal tracts. **A.** Schematic illustration showing the axonal morphology, with highlights of the blue-colored primary axonal tract, which is a long projecting axonal path after excluding short segments at its tip. A neuron may contain multiple tracts, such as the secondary tract highlighted in dark orange. **B.** Schematic visualization of three distinct projection patterns at the population level: convergent, divergent, and parallel, determined based on the comparative spread in space of somas and terminals. The convergent pattern is characterized by a high degree of aggregation in the projection termini. The divergent and parallel patterns are similarly defined, except that their termini regions have larger or similar spread, respectively, compared to their soma regions. Soma positions are indicated by red dots, while arrowheads denote the terminal points of primary axonal tracts. The blue lines connecting them represent the primary axonal tracts. **C.** 2D projections of primary axonal tracts of 25 projection-based subtypes in three brain areas: cortex, striatum, and thalamus. The label on the left specifies the s-type (for CP neurons) or projection classes. Circular red dots represent the somas, while triangular black dots denote the tract termini. In-between tracts are colored randomly. A line plot of the spatial spread (radius) change from the somas to the terminals along the corresponding tracts is appended on the right side for each project type. **D.** Horizontal view of projection pattern maps by source (left) and target (right) regions. The regions are colored by the projection pattern type. **E.** 3D scatter plot of the terminal point locations for three clusters identified for the L5 ET projecting cortical SSp-m neurons using K-Means clustering based on their terminal points, with the respective spatial spread profiles plotted on the right. The terminal points of the three classes are colored in red (C1), green (C2), and blue (C3).

Based on the evidence that fully reconstructed neuronal morphology aligns with neuron class (Figure 4), we further investigated neurons innervating multiple brain regions based on (a) the arborization patterns for both dendrites and axons (Figure 5), and (b) the fiber-projecting patterns that connect these arbors (Figure 6).

Sub-neuronal arbors are dense branching sub-trees of full neuron morphologies. Practically the diameter of an arbor can range from about 100 micrometers to millimeters (Figure 1D). Such dense branching packed tightly in space may be indicative of putative structural and functional units. Thus, profiling the level of arbor stereotypy can reveal information that adds on that inferred from full morphologies. We decomposed a single neuron into a series of arbors to obtain the sub-neuronal representation. The basal and apical dendrites were treated as independent arbors due to their obvious layout. We used an AutoArbor algorithm (Peng et al., 2021; Methods) to divide axons into multiple internally connected arbors. To facilitate comparison, axons of neurons in the same brain region were decomposed to have the same number of arbors, which was determined using the majority-vote method for all neurons in the region. Two kinds of arbors, proximal and distal, were defined based on distance from the soma using a threshold of 750 μm (Figure 5A). The arbors were sequentially ordered by their Euclidean distances to soma, e.g., A1, A2, A3 (if there was one). Following this method, we detected 3,803 axonal arbors, 1,891 basal dendritic arbors, and 625 apical dendritic arbors. We considered a number of morphological features (Methods) specially designed for the arbor structures, e.g., arbor type (proximal or distal), the volume of the rotated 3-D bounding box of the arbor (μm^3^), the number of branches, and the Euclidean distance to the soma (dist2soma).

We analyzed arbor features in three brain areas: thalamus, cortex, and striatum. Quantitative analyses showed that morphological diversity and stereotypy among these 3 areas, including 20 CCFv3 regions each with at least 10 reconstructed neurons, were reflected in multiple aspects, particularly for axonal arbors (Figure 5A). Overall, neurons in the cortex and striatum have around 50% proximal arbors, while thalamic regions have an apparently smaller number of proximal arbors. The extent of proximal arbors is also considerably variable in the thalamus, i.e., VPL and VPM have more proximal arbors than other thalamic regions. The branching number and the respective maximum density features are consistent with arborization patterns revealed mostly by the arbor-volume feature, which indicates that several neurons originating in multiple cortical regions have very large arbors. AId, SSp-n and MOs neurons have a clearly larger axonal arbor A2 than neurons in other regions. MOp have smaller axonal arbors A2 than MOs. By contrast, SSs neurons have only one large axonal arbor A1, which also has a chance to position beyond or below the 750 μm threshold to be either a distal or a proximal arbor. Remarkably, brain regions in the SSp area display dramatically contrasting and indeed combinatorial arborization patterns. SSp-ul have comparable arbors A1, A2, and A3; however, SSp-m, SSp-n and SSp-bfd have large A2 arbors while SSp-ll neurons prefer to have a large, distal axonal arbor (A3).

These arborization patterns of cortical neurons, particularly SSp neurons, seem to define a “codebook” that we sought to further examine. We compared arbors of two major cortical projection classes, i.e., extratelencephalic (ET) and intraelencephalic (IT) neurons (Figure 5B). Differences between projection classes are evident in the dendritic features. Indeed, ET neurons have both larger apical and basal dendrites than IT neurons residing in the same brain regions. However, compared to ET neurons, IT neurons have higher maximum compartment densities for basal dendrites, but lower maximum compartment densities for apical dendrites. In contrast, for axonal arbors, ET neurons have comparable A1-arbor layout with IT neurons, but a greater chance to have a larger A2 than the respective IT neurons, consistent with the categorization of these ET-IT neurons.

We also examined the features of neurons in six regions of the primary somatosensory cortex across cortical layers (Figure 5C). Neurons in the barrel field (SSp-bfd) have large proximal axonal arbors projecting mainly to the cortical layer 6 (L6), but not to layer 1 (L1), layer 2/3 (L2/3), and layer 4 (L4), and distal arbors mainly projecting to L4 and L5. Interestingly, when we subdivided the neurons by laminar position, the projection patterns of proximal and distal arbors had their own attributes, but also exhibited overlaps. Axonal arbors of L2/3 neurons primarily project to L2/3 and L5, while L4 neurons reach mostly L2/3. Instead, L5 neurons project mostly to L5 and L6, and L6 neurons extend projections preferentially to L5 (Figure 5C). All these combinations may be viewed in the circulated visualization with both soma regions and cortical layer information displayed (Figure 5C - circular view). Importantly, we caution that this codebook might change as more neuron reconstructions become available.

As we observed that thalamic neurons have a variety of arborization patterns (Figure 5A), we clustered both the morphological features of arbors (8-dimension) and projection distributions (108-dimension) of neurons originating from each brain region (Methods). We found that thalamic core and matrix neurons have similar projection volumes overall (Figure 5D). In detail, matrix neurons from RE, LD and VM have greater variability of the projection volume than neurons originating in other regions. Morphologically, axonal arbors of thalamic matrix neurons are generally larger, and their structures are more complex, exhibiting a greater diversity than thalamic core neurons (Figure 5D). Indeed, arbors of thalamic core neurons, except LGd, are more conserved in both branching and volume. In terms of projections, thalamic core neurons have a higher concentration of arbors in mostly cortical and midbrain areas, which are responsible for sensory and motor control. On the other hand, thalamic matrix neurons have a wide range of projection targets, covering a larger number (108 out of 314 in our data) of target regions.

### Characterizing motifs of primary axonal tracts

Complementary to the analysis of neuronal arborization, we further studied the projecting axonal fibers connecting major arbors (Figure 6A). The diversity and stereotypy of axonal tracts may help to understand the global structure of the brain. We focused on the primary axonal tracts, which are extracted by iteratively pruning short branches off the longest axonal path (Figure 6A, Methods). We identified three projection patterns, i.e., convergent, divergent, and parallel (Figure 6B).

For 19 major brain regions that contain fully reconstructed neurons SEU-A1891, we found very different projection patterns (Figure 6C). First, stratal and thalamic neurons demonstrate opposite tendencies in projection. SNr-projecting CP neurons (CP_SNr) and GPe-projecting CP neurons (CP_GPe) have clearly convergent patterns, as their somas are widely distributed but the primary projection targets are proximal. The respective cross-sectional radii tend to drop from 1.5 mm to sub-millimeters. In contrast, both the thalamic matrix neurons (TH_matrix) and thalamic core neurons (TH_core) show an evident divergent pattern, as their somas concentrate in all the eight thalamic regions, i.e. LP, VM, LGd, MG, SMT, VPL, VPLpc, and VPM, but the respective projection targets are significantly spread. The cross-sectional radii go northeast from sub-millimeter to about 1.5 millimeters for the TH_core and also VM neurons, and more dramatically to the range of 2~3 millimeters for LP neurons.

Different from the striatum and thalamus, the cortical neurons show more complex patterns (Figure 6C). IT-projecting cortical neurons (CTX_IT) display divergent projections, expanding the cross-sectional radii about 3 times or more over the length of the primary axonal tracts. However, ET-projecting cortical neurons (CTX_ET) have primary projecting axons traveling in a much more conserved way than CTX_IT neurons from somas to the target brain regions. The axon projections of CTX_ET neurons only deviate near target regions.

We mapped all these conserved projection motifs onto CCFv3, with both soma regions and the project target regions highlighted (Figure 6D). Based on our current data, the brain-wide axonal projects are heavily divergent, regardless of the locations of somas, except for occasional cases like CP-SNr and CP-GPe. However, it is also remarkable to see that the divergent CTX_ET projections can be further factorized in terms of clustered target brain regions (Figure 6C - CTX_ET row). For instance, CTX_ET SSp-m neurons have divergent projections, but their targets can be grouped into three clusters (Figure 6E, Supplementary Figure S7). When we separated these targets and profiled the respective projection patterns, they could be either convergent or weakly divergent, and had a parallel pattern when only these targets were considered. In other words, the cortical neurons may have a strongly stereotyped, target-dependent projection pattern although overall the diversity is visibly dominant. In this way, these stereotyped projection motifs provide a high-level description of neuronal arbors across the entire brain.

### Cross-scale topography of axonal boutons

**Figure 7.**
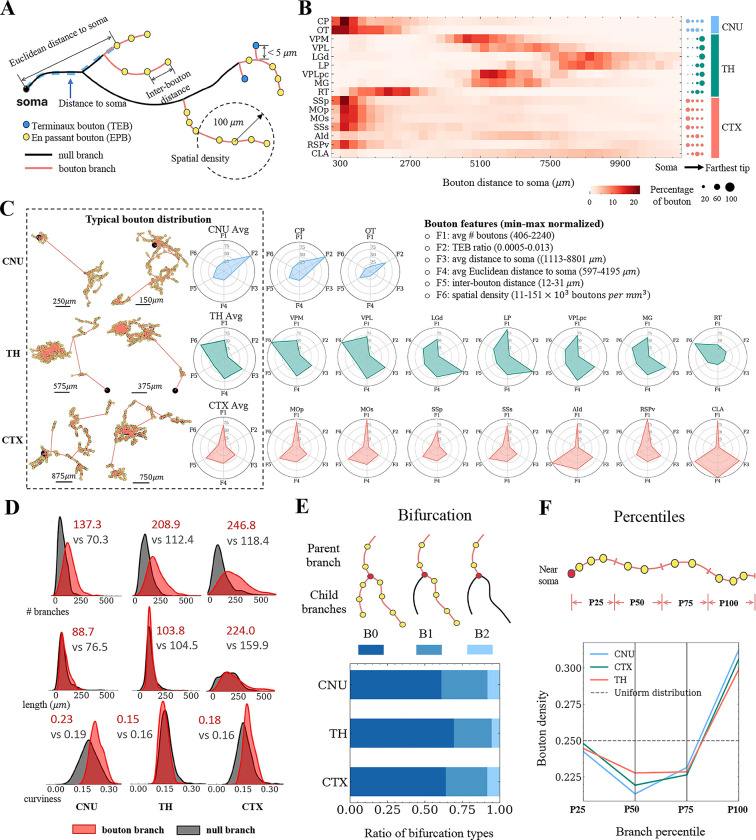
Spatial patterns of bouton distribution at various scales. **A.** A schematic image depicting bouton types and key features of boutons. Specifically, inter-bouton distance represents the axonal length traversed from a bouton to its preceding bouton towards soma. **B.** Heatmap of the percentage of boutons as a function of the distance to the soma (x-axis). Each row is a cell type defined by its soma location (s-type). The percentage is calculated by dividing the number of boutons in each distance range by the total number of boutons for each s-type. The right panel shows the distribution of boutons is represented by categorizing the distance to the soma into four percentiles. The distances for each s-type are normalized independently by the maximal distance among all boutons for the corresponding s-type. The labels on the right specify the brain areas each s-type belongs to. **C**. Within the dashed line frame: graphs on the left side show representative neurons from the cerebral nuclei (CNU), thalamus (TH), and cortex (CTX), with somas (black markers) and boutons (yellow markers) connected by a minimum spanning tree (MST). On the right side, the radar charts show the average of six bouton features. The feature ‘avg # boutons’ represents the average total number of boutons per neuron, and the ‘TEB ratio’ is the ratio of *terminaux* boutons. The other four features are illustrated in Panel A. Each feature value is calculated as the mean for distinct categories and then min-max normalized to scale the values in the 0–100 range. Right panel: Analogous radar charts for each of the s-types within the analyzed brain areas. **D**-**F**. Spatial preference of boutons at various sub-neuronal scales. **D**. Density plots of three morphological features between bouton branches (red, branches containing boutons) and null branches (gray, branches without boutons) among neurons from CNU, CTX and TH. The feature ‘length’ refers to the path length of a branch, while ‘curviness’ represents the curviness of the branch, defined as 1 minus the Euclidean distance between the starting and terminal points divided by the path length. The colored numbers are the mean values of the corresponding categories. **E**. Top: Schematic drawing of three bifurcation types defined according to the presence of boutons in the two child branches. The parent and child branches are topologically connected, with the parent branches being closer to the soma. Bottom: Barplot of the proportions of the three types of bifurcations in each analyzed brain area. **F**. Top: Schematic drawing of the length quartiles of a bouton branch. Bottom: Line plot of the ratio of boutons distributed at quartiles of a bouton branch. The horizontal dashed line represents the expected distribution if boutons were uniformly spaced.

After estimating axonal and dendritic arborizations, we sought to identify putative synaptic sites. Since dendritic spines were not clearly labeled in IMG205, we decided to focus on putative axonal varicosities or boutons. There are two types of boutons: *terminaux* bouton (TEB) and *en passant* boutons (EPB) (Figure 7A) (Anderson & Martin, 2001). By utilizing the complete axons in SEU-A1891 neurons, we identified both types of boutons. To maximize accuracy, we refined the manually annotated skeleton of neurons using an automated skeleton de-skewing algorithm (Li et al., 2023), followed by approximating boutons using a Gaussian distribution model (Methods; Supplementary Figure S8). We identified 2.58 million axonal boutons in total for SEU-A1891, or 1,363 boutons per neuron. We also categorized axonal branches into bouton-branches or null-branches, based on the presence or absence of putative boutons (Figure 7A).

We studied the spatial distributions of boutons at several scales. At the whole-neuron level, we calculated bouton densities as a function of their distances to the respective somas in 16 brain regions (Figure 7B). Boutons of thalamic neurons are predominantly located on the distal axons. CLA and AId neurons have very broad bouton distributions. Olfactory tubercle (OT) and RT neurons also have high bouton density along intermediate range of their axon extensions. Neurons in the other brain regions, including the striatal region CP and 5 cortical regions, have enriched boutons in local axons (Figure 7B).

We also generated a bouton-feature topography for different neurons (Figure 7C). In each of three major categories of brain areas (cerebral nuclei (CNU), thalamus, and cortex), the 6 different types of features of bouton distributions are tipically stereotyped, with the exception of RT neurons which have a different feature map from the other thalamic neurons. Cerebral nuclei (CNU) neurons, particularly caudoputamen (CP) and olfactory tubercle (OT) neurons, are featured with much higher *terminaux* bouton ratios. However, the average patterns of these three brain areas are diversified, offering more detail than the one-dimensional radial distributions (Figure 7B) that are also summarized as the third bouton feature F3 (Figure 7C).

In our data, neurons from cortical regions have 246.8 bouton-branches and 118.4 null-branches on average (Figure 7D). Neurons from the striatum and thalamus regions share a similar property, i.e. the number of bouton-branches almost doubles the number of null-branches (Figure 7D). Higher bouton-branch ratios were found in terminal branches than in bifurcating branches such as 80% of the former containing boutons versus only 53% of the latter. Interestingly, the average lengths of bouton-branches and null-branches are indistinguishable (Figure 7D). On average, bouton-branches of striatum neurons are slightly more curved than null-branches (Figure 7D). At the branch level, we categorized each with-bouton bifurcating branch into three types depending on the type of children branches (Figure 7E). There is a dominance of consecutive bouton-containing branches (B0 and B1 types, Figure 7E). These observations suggest that boutons may aggregate at close-packing axonal arbors. We also found clear differences in the number of boutons at the individual branch level for various neuron types (Figure 7D). Furthermore, axonal boutons are preferentially located at the terminal ends of the branch and are less frequent in the middle of a branch (Figure 7F). Overall, our data suggest that bouton distribution strongly depends on the scale of analysis: while the spatial layouts of boutons vary dramatically at the full neuron level (global diversity), they tend to share analogous patterns at lower structural levels (local stereotypy).

### Characterizing whole-brain diversity and stereotypy using cross-scale features

**Figure 8.**
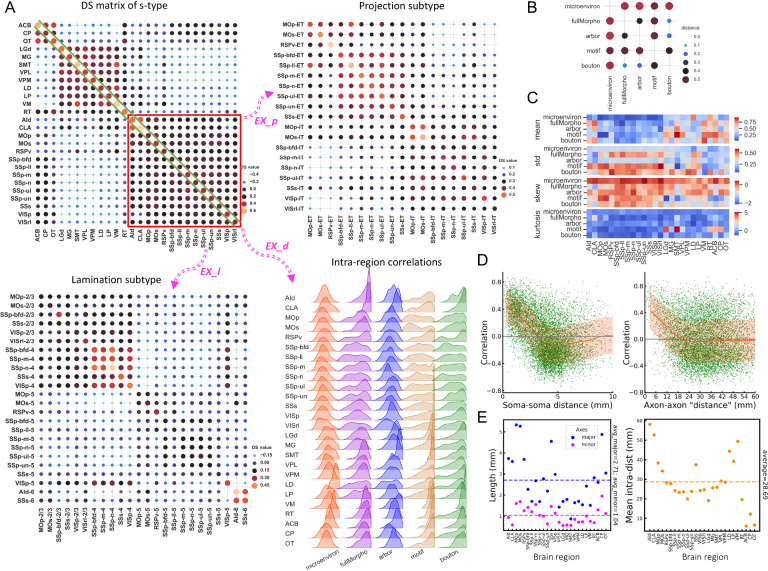
Quantitative diversity and stereotypy analyses based on cross-scale features. **A.** The Diversity-and-Stereotypy matrices (DS matrices) for s-types (upper left), projection subtypes of cortical neurons (upper right), and lamination-based subtypes of cortical neurons (bottom left). 26 s-types were evaluated, including 14 cortical types (AId, CLA, MOp, MOs, RSPv, SSp-bfd, SSp-ll, SSp-m, SSp-n, SSp-ul, SSp-un, SSs, VISp, and VISrl), 9 thalamic types (LGd, MG, SMT, VPL, VPM, LD, LP, VM, and RT), and 3 striatal types (ACB, CP, and OT). The nomenclature used for these types follows the CCFv3 atlas. Each value in the matrix (DS value) is the average correlation between all neuron pairs of the two corresponding cell types. The diagonal values are the intra-region average correlations, and the others are inter-region average values, representing intra-region stereotypy and inter-region diversity respectively. The correlation is the Pearson correlation coefficient between cross-scale features, which are the concatenation of standardized features from 5 morphological scales: microenvironment (‘microenviron’), full morphology (‘fullMorpho’), arbor, primary axon tracts (‘motif’), and bouton. Bottom right: Density plots of the distributions of intra-region correlations for various s-types at different morphometry scales, that is, the distribution of diagonal items in the left component of panel A. **B**. Pairwise distances between the DS matrices of different scales. The distance is obtained by computing 1 minus the Pearson correlation coefficient of the DS matrices. **C**. Heatmaps of the first, second, third, and fourth orders of statistics of the intra-region correlation distributions for each morphological scale (bottom right of panel A). **D**. Scatter plots showing the relationship between the spatial profiles, including soma-soma distances (left) or axon-axon “distance” (right), and the correlations of the cross-scale morphometry features. The axon-axon “distance” is the Euclidean distance between the projection vectors of the neuron pair. The whole-brain projection is defined as a region-wide axon length vector that contains the total axon length for all regions in millimeters. Linear correlations are observed for both cases when the pairwise distances are small. The red lines and red shadows within the boxes represent the means and correlation ranges within one standard deviation (σ) around the mean values. **E**. Scatter plot of the major and minor axis lengths of brain regions (left) and the mean intra-distance of axonal pairs within each region. The dashed lines in the left box are the average lengths for the major and minor axes of all regions. The dashed line in the right box is the average distance for all regions.

In observing substantial diversity across different morphometry scales, we questioned whether such diversified patterns across scales could be combined to characterize neurons. To do so, for each neuron, $n_{i}$, we first concatenated its features of all five resolution scales (microenvironment, full morphology, arbors, motifs, and boutons) as a feature vector $f_{i}$. Then, for two neurons $n_{i}$ and $n_{j}$, we used Pearson correlation of the concatenated features of two neurons, $c_{ij}$, to measure the similarity between this neuron pair. Next, for neurons innervated from two brain regions U and V, i.e., two soma-types (s-types), we averaged the correlation coefficients of all inter-region neuron pairs to produce an overall similarity score $s_{UV}$ of the neurons in these two regions based on cross-scale features. A $s_{UV}$ score close to 0 indicates that neurons in these two regions have little in common. A $s_{UV}$ score approaches the upper limit 1 indicates that neurons in the two regions have many common features, while a $s_{UV}$ score approaches the lower limit −1 indicates opposite features. Therefore, $s_{UV}$ measures the diversity of neuronal features across scales and regions. Clearly, when U and V are actually the same region, the score becomes $s_{UU}$ (or $s_{U}$ for simplicity), which measures the intra-region averaged similarity, or equivalently the “intra-type” stereotypy, of neurons. In this way, we constructed a Diversity-and-Stereotypy (DS) matrix S, in which each entry is $s_{UV}$, for all pairs of brain regions to quantify the distribution of neuronal patterns (Figure 8A).

We found that cross-scale features were able to discriminate between different neuron soma-types. Indeed, the DS matrix of all soma-types in this study shows three clear modules, which correspond to the majority of cortical, thalamic, and striatal neurons (Figure 8A - top-left), except the thalamus reticular nucleus (RT) neurons, which are distinguishable from other thalamic neurons in terms of neurotransmitters and connectivity (Roy et al., 2022). In addition, the DS submatrix of cortical neurons correlates negatively with that of the thalamic neurons, but exhibits weak correlation with the striatal neurons. Thalamic neurons also correlate weakly but also negatively with the striatal neurons in the DS matrix. Within each module, the DS values are relatively large but have small variations, indicating that neurons are remarkably conserved in these brain regions. This grouping of brain regions based on cross-scale features is also consistent with our alternative analyses, e.g., microenvironment analysis (Figure 3).

We focused on the diagonal of the DS matrix (Figure 8A - *EX_d*) to examine the distribution of features for the five resolution scales (Figure 8A, - intra-region correlations, Supplementary Figure S9). Although overall cortical, thalamic, and striatal neurons have similar average DS scores within each brain region (mean-values = 0.36, 0.48, and 0.47, respectively, as shown as the diagonal values in Figure 8A - DS matrix of s-type), they have different degrees of stereotypy with respect to morphological scales. For instance, for microenvironment features, the average correlation value of thalamic neurons (0.44) is much larger than that of cortical neurons (0.08) (Figure 8A - Intra-region correlations), indicating that microenvironment features would be more discriminating for thalamic neurons than for cortical neurons. Similarly, we found cortical neurons in certain regions could be characterized by alternative morphological scales. One example is CLA neurons, which have highly conserved full morphology and bouton features, as indicated by the high mean value of the correlation (0.8 and 0.7, respectively) (Figure 8A - Intra-region correlations).

We also used the DS matrix to examine subtypes of neurons. We focused on two subtypes, i.e., neuron-projection subtypes (Figure 8A - *EX_p*) and soma-lamination subtypes (Figure 8A - *EX_I*) for cortical regions that contain at least 10 fully reconstructed neurons in our data. For the projection subtypes (Figure 8A - *EX_p*), most DS scores among ET neurons are larger than 0.3, which also holds true for IT neurons. However, the majority of ET neurons correlate weakly with IT neurons, even when they are located in the same brain regions (e.g., SSp-n-ET vs SSp-n-IT neurons). Interestingly, several projection subtypes such as MOp-IT, MOs-IT, SSs-IT, and SSs-ET neurons show considerable correlations with all neuron subtypes. The DS matrix also highlights an interesting submodule composed of six SSp ET projecting subtypes, with pairwise correlations higher than 0.4 in most cases.

Modularity was also observed for cortical laminar subtypes (Figure 8A - *EX_I*). L2/3 and L4 neurons are inter-correlated with each other, but exhibit weak correlation with other layers. The boundaries of modules are clear. In the module of L2/3-L4 neurons, a sub-module consisting of five L4 subtypes, SSp-bfd-4, SSp-m-4, SSp-n-4, SSs-4, and VISp-4, also stands out, with a DS score around 0.4. L5 subtypes also appear stereotyped in the DS matrix, but the inter-region correlation tends to be weak, in the 0.15 range. The two L6 subtypes, AId-6 and SSs-6, highly resemble each other but have slightly different correlation profiles with other subtypes. Interestingly, VISp-5 neurons show negative correlations with most of the L5 neurons and all L6 neurons, but correlate considerably with L4 and L2/3 neurons. In addition, neurons from the same brain region but in different layers are not necessarily correlated. For instance, the L5 subtypes of SSp neurons and the respective L4 subtypes are negatively and weakly correlated.

We also attempted to understand the relationship among features of different scales. To do so, we calculated the “distance” between each pair of scales (Figure 8B, Methods), along with the statistics of these features for different brain regions (Figure 8C). We found that microenvironment and motif features were far away from features of other scales, but bouton features had small distances to both full morphology and arbor features (Figure 8B). Therefore, the microenvironment and motif features have relatively little redundancy when they are combined with other scales to categorize neurons and brain regions, while the two separate pairs of scales, i.e. {full morphology and bouton}, {arbor and bouton} could be used to cross-validate whether or not data analyses are consistent across scales.

Our analysis above, especially the DS matrices of the projection and lamination subtypes of cortical neurons, indicate that neuronal types could be well defined by their axonal projections and soma location (Figure 8A). It also suggests an underlying relationship between spatial distribution and morphogenesis, i.e., proximal neurons sharing more similar morphologies. We tested this hypothesis by evaluating the relationship between the morphological correlation of neurons and spatial distance, including soma-to-soma distance and axon-to-axon “distance”. The morphological similarity between neurons was linearly correlated with both the soma-to-soma distance and axon-to-axon “distance”, within a scale at 4 millimeters and 24 millimeters respectively, which are at comparable sizes of brain regions (Figure 8D–E).

## Discussion

In this study we studied the morphological patterns of neurons in the context of whole mouse brains at multi-scales, from centimeters to sub-microns, with specific focus on the quantification of the diversity and stereotypy of neuronal structures. We leveraged the collaborative effort of the BICCN community to collect and standardize one of the largest mammalian brain imaging databases to the latest Allen Common Coordinate Framework, followed by systematic extraction of morphological features from whole brain level to axonal bouton level. Subsequently, we categorized neuronal patterns in the cortex, striatum, and thalamus, in conjunction with their soma-distribution, projection trajectories and targets, and more detailed arborization and putative axonal boutons when applicable. Using rich representations of morphological data, we discovered brain modules and morphology motifs across scales, and identified the suitable spatial scales for quantifying the diversity and stereotypy of neuronal patterns.

Our multi-scale analysis is unique, complementing a number of previous efforts in generating macroscale, mesoscale, and microscale morphometry in the mouse brain (Shapson-Coe et al., 2021; Cabral et al., 2023; Oh et al., 2014; Harris et al., 2019; Whitesell et al., 2021; Witvliet et al., 2021).At the neuron-population level, we analyzed the modular organization of brain regions based on neurite distribution patterns. Previously, modules of mammalian brains have been studied in macroscale, primarily using functional Magnetic Resonance Imaging (Bertolero et al., 2015), and in mesoscale, such as the brain-wide neuronal population based projecting-networks using whole-brain optical imaging (Harris et al., 2019; Oh et al., 2014; Benavidez et al., 2021). Our analysis confirmed several previous observations such as neighboring regions being more likely in the same module (Bertolero et al., 2015; Harris et al., 2019). We also additionally estimated modularization from large-scale analysis at the micron and even sub-micron resolutions.

We constructed dendritic microenvironments to enhance the ability to discriminate the structure of local dendrite arborization. Historically, the morphological features of local dendrites were thought to offer limited power for discriminating neuronal classes (Gouwens et al., 2020; Kozareva et al., 2021). These observations have also motivated recent studies that rely on fully reconstructed long axons to differentiate neuron classes (e.g. Winnubst et al., 2019; Peng et al., 2021; Gao et al., 2022). Nonetheless, the cost to produce long axons or full neuron morphology is still high, and sometimes is exceedingly expensive for large mammalian brains such as primates (Ascoli et al., 2022). We have recently proposed aggregating the spatial neighboring information of local dendrites of human cortical neurons with their 3-D morphology, and thus have obtained superior classification performance of neurons (Han et al., 2023). In this study, we followed the same principle to formulate dendritic microenvironments that offer a valid alternative to integrate spatial information of neurons and their morphology. Our approach has allowed visualization of more anatomical detail for several brain regions compared to what had been documented in the CCFv3 atlas (Wang et al., 2020) and the Mouse Brain in Stereotaxic Coordinates (Paxinos & Franklin, 2019).

In addition to introducing dendritic microenvironments, we were able to identify critical, minimally redundant factors that contribute to the different categorizations of individual neurons, for their full morphologies. We found that the clustering of cortical, striatal and thalamic neurons into broadly recognizable clusters, each with a specific fingerprint, could emerge with little a priori knowledge. The key features could be identified in the least redundant subspace of spatially tuned morphology features. This finding also complements the conventional parcellation of brain regions in anatomical atlases primarily based on cell densities. Future studies in this direction, potentially combined with the microenvironment analysis of neurites, might suggest alternative approaches to investigate the murine brain anatomy using morphological, physiological, molecular and connectional properties of neurons (Zeng & Sanes, 2017; Miller et al., 2020).

Individual neurons have traditionally been studied by analyzing their overall morphology (e.g. Wan et al., 2015; Gao et al., 2023). However, it is intriguing to explore the variability of arborization and projection patterns within neurons, as they naturally constitute interconnected sub-trees and projecting neurite tracts. We note that this aspect has not been extensively investigated to date. To address this, we undertook a decomposition of single-neuron morphologies into densely packed sub-trees, referred to as arbors. These arbors serve as structural foundations for potential neuronal functions. Additionally, we categorized the arbors according to their proximity to the respective somas. Furthermore, we extracted the primary projecting tracts of neurons originating from different brain regions and examined their spatial divergence and convergence patterns. This approach simplifies the comparison of different neuron types while retaining crucial morphological information. Moreover, it facilitates the quantification of the diversity of conserved patterns, denoted as “motifs” of arbors and neurite tracts. Our work complements previous endeavors aimed at characterizing sub-neuronal structures, such as branching topologies (Gillette & Ascoli, 2015; Lin et al., 2018).

The investigation of synaptic connectivity is a contemporary and critical topic. While electron microscopy remains the gold standard for synapse identification, its limited range (~1mm^3^) currently prevents its applicability to mammalian brain-wide axonal projections. Previous studies have thus focused on detecting and analyzing potential synaptic sites collected by optical microscopy (Hallock et al., 2008; Gala et al., 2017; Xie et al., 2017) using various labeling techniques, including genetic or antibody labeling for presynaptic and/or postsynaptic sites, as well as a combination of both (Micheva & Smith, 2007; J. Kim et al., 2012; Iascone et al., 2020). This study aims to expand on existing synapse-detection research in three ways. First, the full morphologies of nearly 2,000 neurons were used to provide a comprehensive dataset for analysis. Second, whole-brains, encompassing a number of cortical, striatal, and thalamic regions, were used to provide a complete picture of the distribution of putative synaptic sites. Third, we explored a wide range of features associated with putative synapses. In this way, we have characterized the patterns of brain-wide bouton-distributions across various cell types that complement previous studies.

The knowledge gathered from investigating various spatial scales prompted us to develop an integrated model of neuron morphometry and brain anatomy. As an initial effort, we introduced a DS matrix to measure the degree of diversity across neurons with respect to the stereotypy of neuron types. We observed interesting hierarchical and modularized organization of neurons in cortical, striatal and thalamic regions emerging in a quantifiable way, even without explicit clustering. This finding has two valuable implications. First, it confirms complex neuron morphology strongly correlates with existing brain anatomy in the established mouse brain atlases such as CCFv3. Second, and more importantly, it allows us to hypothesize that for a more complicated mammalian brain such as those of primates, an effective way to explore and understand the brain anatomy and even the associated brain functions could take a similar multi-scale approach, instead of relying solely on anatomists’ manual drawing of brain structures. The present study highlights the power of large scale systematically mapped neuronal data in elucidating detailed cell type structure and morphology. Our cross-scale, multi-modality integration of information may also extend to incorporate in the future other data modalities such as single-cell transcriptomic data (Gouwens et al., 2020; Yao et al., 2021; Allen et al., 2023).

## Methods

### Nomenclature for brain regions and areas

All anatomical regions and their hierarchy follow the CCFv3 nomenclature (Wang et al., 2020), which segments a mouse brain into 671 regions, with each region (except for the direct tectospinal pathway, tspd) comprising two mirroring subregions in the left and right hemispheres. A higher level of granularity consisting of 314 CCFv3 regions (CCF-R314) is used by merging highly homogeneous regions, such as the lamination-differentiated cortical subregions. All brain regions used in this work are, unless otherwise stated, from the CCF-R314 regions. We have spelled out the full names of the regions in the manuscript whenever we refer to them for the first time. To access the complete names of CCFv3 regions, please consult the online viewer of the Allen Reference Atlas, which can be found at https://connectivity.brain-map.org/3d-viewer?v=1.

Super-regional anatomical entities, such as brain areas, are sets of functionally or anatomically related regions that are continuous in space and are defined in CCFv3. While the definitions of brain areas are similar, they differ in granularity. In this paper, we discussed a higher granularity consisting of 4 areas: cortex (CTX), cerebellum (CB), cerebral nuclei (CNU), and brain stem (BS). We also discussed 13 compound areas, which are CBN: cerebellar nuclei, CBX: cerebellar cortex, CTXsp: cortical subplate, HPF: hippocampal formation, HY: hypothalamus, isocortex, MB: midbrain, MY: medulla, OLF: olfactory areas, P: pons, PAL: pallidum, STR: striatum, and TH: thalamus.

### Image acquisition and processing

We collected a total of 205 whole mouse brains at submicron or micron resolutions from 4 BICCN projects within the BICCN community, and another collaboration project. Of these, 181 fMOST brains came from a U19 project (1U19MH114830-01). The other 10 fMOST brains and 10 STPT brains were obtained from another U19 project (1U19MH114821-01) and 1 LSFM brain from a U01 project (1U01MH114829-01). All of these brains were downloaded from the Brain Image Library (BIL, http://www.brainimagelibrary.org). 3 LSFM mouse brains were provided by P.O. (n=2) and Z.W. (n=1), who were granted from another U01 project (1U01MH114824-01). All of these brains have anisotropic resolutions, with most brains having a resolution of 0.2–0.35 μm in the xy plane and 1 μm in the z direction. Meta information, including their brain IDs, modalities, sources, resolutions, downloadable links, etc., is also provided (Supplementary Table S1).

The brain datasets comprise a total of 3.7 peta-voxels and are managed with a centralized data management system MorphoHub (Jiang et al., 2022), in which the data is accessible from multiple clients, including local workstations, supercomputers, immersive headsets, web interfaces, and our mobile platform Hi5, through high-speed networks. To get fast access to both fine-grained details and a global overview, we restructure each brain into hierarchical TeraFly (Bria et al., 2016) format.

### Registration

Brain images were registered to the 25 μm CCFv3 template using the cross-modal registration tool mBrainAligner (Li et al., 2022; Qu et al., 2022). We followed a similar pipeline as described in these papers, with a minor update on the landmark searching strategy which improved overall registration accuracy by approximately 50%, particularly for the hippocampal and striatal neurons. Registration channels were leveraged whenever possible. The brains were down-sampled to approximately 25 μm resolution through even-folds linear interpolation prior to registration. Non-brain tissues were semi-automatically removed with Vaa3D (Peng, Ruan, Long, et al., 2010; Peng et al., 2014; Liang et al., 2023) and mBrainAligner. Anatomical regions of the brains were automatically labeled based on the CCFv3 atlas and the deformation matrices obtained during registration. The multi-morphometry including full morphologies, local morphologies, arbors, and axonal boutons, were reverse-mapped to the CCFv3 space using the inverse deformation matrix.

### Soma identification

The SEU-S227K soma dataset comprises two parts. The first part consists of 51,945 somas that were manually annotated using the Vaa3D-TeraFly platform, most of which were reported in a previous study (Jiang et al., 2022). An additional 175,636 somas were semi-automatically annotated using our updated multi-morphometry visualization and annotation platform, Collaborative Augmented Reconstruction (CAR) (Peng et al., 2023, unpublished).

The semi-automatic soma identification protocol involves two major steps. Firstly, the highest-resolution whole-brain images were divided into blocks of approximately 256 voxels in each dimension. In practice, we directly utilized the highest-resolution TeraFly image blocks which complied with our desired block size. We then filtered out the blocks with maximal intensities less than 250 (unsigned 16-bit image), standardized the remaining blocks through a Z-score normalization, and converted them to the unsigned 8-bit range. Next, the blocks were binarized using their 99th percentile as thresholds, and the resulting images were transformed using the gray-scale distance transform (GSDT) algorithm. Voxels with intensities in the range of 5 to 30 on the transformed image were identified as candidates, which were further processed using a Non-Maximal-Suppression (NMS) like approach to eliminate redundant candidates.

In the second step, we cropped 128×128×128-sized image blocks centered at the position of putative somas on the second-highest resolution images, as a compromise between efficiency and accuracy. These image blocks were then distributed to remote users on the mobile application Hi5. Using this protocol, we were able to identify 179,115 somas within weeks, with the involvement of 23 trained annotators and 7 fresh annotators without any prior knowledge.

The soma locations were then optimized by applying the mean-shift algorithm bound with Vaa3D after zero-clipping of voxels with intensity lower than μ+σ, where μ and σ are the mean and standard deviation of the soma block. A window size of 15 voxels was used for the mean-shift soma location optimization. Possible duplicates were removed when two somas are within 15 voxels and their center point intensity is lower than the average intensity of the two somas. Applying these post-processing steps yielded a soma dataset comprising 51,945 manually annotated somas and 175,636 semi-automatically annotated somas, which together constitute the 227,581 soma dataset (SEU-S227K).

### Neurite signal estimation

Neurite signals were segmented using an automatic algorithm and then summarized the number of voxels by brain regions to produce a region-wide signal vector consisting of 314 values (regions). Each value represented the total number of detected neurite voxels in the corresponding region in CCF-R314, and it was then divided by the total number of neurite voxels in the brain. Our algorithm succeeded in detecting signals in 191 out of the 205 brains on low resolution images, averaging at approximately $2\;\mu m\;\left(x\right)\times 1\;\mu m\;\left(y\right)\times 4\;\mu m\;(z)$, for a trade-off between accuracy and efficiency. Among these, 177 brains were from 35 genetically labeled lines (Supplementary Figure S3). Consequently, each of the 314 regions was represented by a brain-wide neurite signal distribution vector, resulting in a neurite density matrix $\left(M_{d}\right)$ with dimensions of 314×191. Using the matrix, we estimated a pair-wise regional correlation map $\left(M_{c}\right)$ with dimensions of 314×314, among which each value was the Spearman correlation coefficient between the normalized 191-dimensional signal vectors of the corresponding region.

Based on the correlation map $\left(M_{c}\right)$, we assessed the intra-compound area consistency as the distribution of correlations between all pairs of regions within the compound area. In this way, the distributions for all 13 compound areas were calculated (Figure 2A).

The neurite segmentation algorithm can be summarized in 6 steps:

1. Estimation of an empirical foreground threshold for each brain. This step involved finding an empirical threshold value (*thresh*) to distinguish between the foreground (neurite signal) and background (non-neurite voxels) on the lowest-resolution image. The threshold was estimated based on the mean and standard deviation of all voxels: 0.9×min(max(μ+1.5σ,400),1000), where μ and σ were the mean and standard deviation of all voxels of the brain (16-bit image).
2. Split the brain into non-overlapping image blocks. In this step, the third lowest resolution brain images were split into small non-overlapping blocks for subsequent memory-affordable processing. We utilized the image blocks of Vaa3D-TeraFly files at the specific resolution of approximately 256 voxels in each dimension.
3. Pre-filtering. This step involves filtering out blocks that are unlikely to contain signals. Image block files (in compressed TIFF format) that were smaller than a certain size (1.7 MB) or had a maximum pixel value lower than 300 were considered non-signal blocks and were excluded.
4. Calculation of the salient map. An image block was firstly denoised by an adaptive filter similar to the ‘ada_threshold’ plugin on Vaa3D. At the same time, an anisotropic salience map was estimated through block-wise PCA analysis on 16×16×16 voxels-sized cuboids which were upsampled from 16×16×4 cuboids of the original image, as the resolution in z axis of the image is about 3 times smaller than that in x and y axes. The anisotropy score of each cuboid is defined as $\frac{S_{1}-S_{2}}{S_{1}+S_{2}}\cdot \frac{S_{1}-S_{3}}{S_{1}+S_{3}}$, where $S_{1},$ $S_{2}$, and $S_{3}$ are the eigenvalues of first, second, and third principal components of the cuboid, similar to the content index Q in previous studies (Liang et al., 2017). The final salient map was calculated by multiplying the denoised image and anisotropy map.
5. Thresholding. The salient map was thresholded using 0.1 × *thresh* calculated in step 1. *thresh* may be manually adjusted based on the segmentation results if it was inappropriate.
6. Mapping neurite voxels to CCFv3 atlas. Finally, the identified neurite voxels were mapped to the CCFv3 space and summarized by regions to obtain a region-wide neurite signal vector in the shape of 314. The vector was then normalized by dividing it by the total number of neurite voxels, resulting in a neurite density vector for the brain.

Our neurite detection algorithm was qualified by the good linearity between the number of annotated somas and the total number of detected neurite voxels of each brain (Supplementary Figure S3D). The considerable variety of sparse labeling and a large number of transgenic lines (n=35) and brains (n=191) provide a minimal-redundant signal matrix, laying the foundation of a reliable neurite detection. Of note, the regional signals are a mixture of in-house soma-centered dendrites and bypassing neurites, which was confirmed by the low linearity between the number of neurite voxels and number of somas of each brain (Bottom of Supplementary Figure S3D). Yet, a large set of diverse labeling genes and brains down-weights the co-expression patterns and highlights the functional relationship. When two regions show a high correlation between their distributions across 191 brains, it may indicate a possible functional connection.

### Target-correlated regions detection

We took each of the 314 CCFv3 regions as the target region and extracted regions whose cross-brain neurite density vector had a Spearman correlation coefficient of no less than 0.8 with it, thereby forming its highly correlated region set. Out of the 314 regions, a total of 79 regions were identified to have highly correlated regions based on the criteria. A detailed list of these region sets is provided in Supplementary Table S4. We classified the region sets into two categories: intra-compound area (intra-CA) and cross-compound area (cross-CA), based on whether the regions were within the same compound area.

### Regional module detection

We classified all regions into 31 non-overlapping subsets or initial modules, based on the dendrogram produced by applying hierarchical clustering to the correlation map $\left(M_{c}\right)$. The module detection process begins by finding a seed branching point, which is the cluster with the lowest level (*i.e.*, the first diverging cluster) in the dendrogram, followed by checking the number of all its subsidiary region leaves. If a cluster contains no less than 3 regions and no more than 15 regions, it is defined as an initial module. Otherwise, we merge the current cluster with the most closing cluster or split it into modules. The process repeats until all regions are categorized into a module, resulting in a total of 31 non-overlapping initial modules.

For all the initial modules, we removed any region that occurred less than twice in the target-correlated regions. If a module had at least two regions remaining, it was considered a tight module. Otherwise, it was deleted. Using these criteria, we identified 16 tight modules (Figure 2B, Supplementary Table S5), each with a high consistency score of no less than 0.57, where the consistency score was calculated as the average Spearman correlation coefficient of the cross-brain neurite density distribution for all pairs in that module.

### Tracing local morphology

The local reconstructions were generated using the somas of SEU-S227K. To avoid highly interweaved neurons, somas with more than five neighboring somas within a radius of approximately 128 μm were eliminated. For each soma, a block measuring 512 × 512 × 256 (x×y×z) voxels was cropped from the second highest resolution images, with the soma located at the center of the block. This block size corresponds to an approximate diameter of 250 μm in xy axes and 500 μm in z axis around the soma, encompassing most of the basal dendrites (98% of total compartments in manual annotations), a significant portion of the apical dendrite (63%), and a few axons.

We combined two automatic tracing algorithms, All-Path-Pruning (APP2) (Xiao & Peng, 2013), and the tubular fitting-based algorithm neuTube (Feng et al., 2015), to trace each image block. Default parameters were used for both algorithms except that the background threshold in APP2 was set to an automatically determined threshold of μ+0.5σ, where μ and σ are the mean and standard deviation of the input image. Reconstructions from APP2 and neuTube were combined to get an initial reconstruction. Specifically, neuTube reconstructions were used to prune the APP2 reconstructions, and nodes in APP2 reconstructions that did not have corresponding nodes within 5 voxels in neuTube reconstructions were pruned.

The generated reconstructions in the previous step were subjected to a segment-pruning pipeline, which rectified possible loops, erratic branches, and intersections with other neurons. Each pruning step operates as an independent filter that takes in the raw neuron tree, and the resulting tree is the intersection of all filtered reconstructions. The detailed pipeline can be summarized as follows:

1. Firstly, branch pruning was performed to remove any branch that had an excessive angle to its parent (<80 degrees) or showed an excessive increase in radius (1.5 times the parent branch’s radius).
2. Secondly, a crossover pruning step was carried out to expunge branches from putative crossover structures. To do that, we detected all putative crossover structures, including multifurcating nodes containing more than two child nodes and consecutive bifurcating nodes within five voxels. For each of the crossover structures, we checked all connections between the current branch and its child branches. In specific, branches with small angles (<80 degrees) were marked as removable. Then, we evaluated branches with mediocre turning angles (80–100 degrees) to confirm if another branch with a large enough angle existed between them (>150 degrees). If such a branch existed, we removed the other branches.
3. Then, a soma pruning strategy was applied to remove branches originating from any other putative somas. A soma candidate was identified when the total area of a candidate node or a set of nearby candidate nodes is large (> 500 pixel^2^ on the maximum intensity projection on the xy plane). A candidate node here is a reconstructed node with a radius larger than five pixels on the xy-plane. Nodes that were too close to the current soma (< 50 pixels) were not checked. For each detected soma, an integration of deviation angle along the fiber path in between the detected soma and the target soma was estimated, to locate the best cutting position. The deviating angle is the angle between a single local branch and the radial line connecting the soma and the nearer end of the same branch, similar to the G-Cut (Li et al., 2019). We then calculate the integral of deviation angles at both sides, that is, from the current branch to the current soma and the current branch to the putative soma respectively, by weighting the deviation angle with the branch length. Branches with a lower deviation angle integral leading to the putative soma were subsequently removed.
4. Next, winding pruning was performed to remove any branch that followed a circuitous path to the soma, defined as the ratio between path distance and Euclidean distance being greater than three.
5. Finally, all subsequent nodes of a pruned branch or nodes identified in the previous steps were removed, and reconstructions with fewer than 20 nodes were discarded.

We reconstructed 15,441 local morphologies following the aforementioned protocol. The morphologies were then mapped to the CCFv3 atlas and automatically labeled the regions of the somas (314 granularity, CCF-R314).

### Dendritic microenvironment construction

A dendritic microenvironment was defined as the spatial-tuned fusion of a local morphology and its top five most similar morphologies within a sphere of radius 249 μm, which was the 50th percentile distance among the distances between the 5th closest neuron and the target neuron for all neurons in SEU-D15K. The similarity between two neurons was calculated using the Euclidean distance of their standardized (Z-score normalized) morphological feature vectors.

Each neuron was represented using a 24-dimensional feature vector consisting of 18 L-Measure (Scorcioni et al., 2008) features (except for the ‘Nodes’, ‘SomaSurface’, ‘AverageDiameter’, and ‘Surfaces’ features of the 22 features described in the ‘Morphology features’ section in the Methods), the explained variance ratios of three principal components (variance percentages of PC_1, PC_2, and PC_3), and sum-normalized values of the first principal component (PC_11, PC_12, and PC_13). The principal components (PC_1, PC_2, PC_3) were calculated using principal component analysis (PCA) for all nodes in isotropic space. The variance percentage of a principal component represents the ratio of its variance among that of all principal components. The microenvironment feature is a spatial proximity weighted averaging of features derived from all six neurons constituting the microenvironment. Specifically, the spatial weight for a neuron is the exponential of negatively normalized distance. Therefore, for each microenvironment, its feature was calculated as:

$$F_{M}=\sum_{i=1}^{6}.w_{i}\times F_{i},\;\text{where}$$

$$w_{i}=\frac{exp\;\left(-d_{i}/D\right)}{\sum_{i=1}^{6}..exp\;\left(-d_{i}/D\right)}$$

where $F_{M}$ and $F_{i}$ are the feature vectors of the microenvironment and neuron i. $d_{i}$ is the distance between the soma of the target neuron and its neighboring neuron i. Here, D is the sphere radius (249 μm).

To intuitively visualize the data, we used the max-relevant min-redundancy (mRMR) algorithm to reduce the 24-dimensional microenvironment feature vector to the three most discriminating features. The identified top three features were straightness, Hausdorff dimension, and variance percentage of the third principal component (PC_3), which represent the branch bending, the fractal dimension of the morphology, and the explained variance ratio of PC_3, respectively. To generate the whole-brain microenvironment map, we initialized an empty image of the same size as the 25 μm resolution CCFv3 atlas and assigned each neuron’s three features to the three color channels (R,G,B) of its soma location in the image. The resulting map contained 15,441 data points that clearly represented the three most discerning microenvironment feature values. Data points located in the right hemisphere were mirrored to the left hemisphere, based on the widely accepted left-right symmetry assumption, which was also validated in this work (Supplementary Figure S4, Supplementary Figure S5>). Each feature underwent min-max normalization and linearly mapped to a range between 0 and 255 for consistent comparison. Additional histogram equalization was applied for visualization purposes, but not for quantitative analysis. Microenvironments located within 1 mm of the middle sections along the axial, sagittal, and coronal views were mapped to the corresponding middle sections using the maximum intensity projection (MIP). The resulting maps were superimposed with the boundary outlines of the CCFv3 atlas to facilitate the semantic analysis of the feature distribution. We employed KMeans clustering to classify regions based on the concatenation of the mean and variance feature vectors of all microenvironments in that region.

### Refining single neuron reconstructions and skeletons

We manually annotated 151 single neurons in their entirety from 20 fMOST mouse brains from the 1U19MH114830-01 project at submicron resolutions. The majority of neurons (n=104) were cortical, with the rest being thalamic and striatal neurons, based on the automatic labeling of brain regions through registration. The annotations were carried out with our Collaborative Augmented Reconstruction system (Peng et al., 2023), which is updated from our Vaa3D-TeraFly and immersive annotation system Vaa3D-TeraVR (Wang et al., 2019). Together with our previously released 1741 neurons (Peng et al., 2021) (excluding one neuron due to quality issues), we got a total of 1891 single-neuron morphologies (SEU-A1891).

The morphologies were annotated at various resolutions, which frequently lead to an image-morphology mismatch in the highest resolution space. To overcome this issue, we developed a retracing strategy to refine the skeletons (Li et al., 2023). This involved a two-step process. First, the skeletons were split into fragments no longer than 50 μm, and every two consecutive middle points of the fragments were connected using the graph-augmented deformable model (GD) (Peng et al., 2010). The GD algorithm automatically fits the skeleton to the nearest salient signals with the constraints of the original skeleton priors. In the second step, an additional step of GD tracing was applied to the middle points of two consecutive refined fragments.

All full morphologies used in this study were based on this refined version of SEU-A1891, among which 1740 had their meta information manually annotated, including regions of cell bodies, projection classes, and cortical laminations. In most experiments presented in this paper, except in Figure 4 and Figure 7, only neurons with manually annotated meta information were leveraged.

### L-Measure features

We considered widely used L-Measure features to characterize the morphologies in several morphometry scales, including microenvironment, full morphology, and local morphology. For consistency, we calculated 22 features implemented in the “global_neuron_feature” plugin of Vaa3D (Peng, et al, 2010; Peng, et al, 2014), namely ‘Nodes’, ‘SomaSurface’, ‘Stems’, ‘Bifurcations’, ‘Branches’, ‘Tips’, ‘OverallWidth’, ‘OverallHeight’, ‘OverallDepth’, ‘AverageDiameter’, ‘Length’, ‘Surface’, ‘Volume’, ‘MaxEuclideanDistance’, ‘MaxPathDistance’, ‘MaxBranchOrder’, ‘AverageContraction’, ‘AverageFragmentation’, ‘AverageParent-daughterRatio’, ‘AverageBifurcationAngleLocal’, ‘AverageBifurcationAngleRemote’, ‘HausdorffDimension’. These features could also be calculated using the L-Measure server (Scorcioni et al., 2008).

### Full morphology analysis

We extracted 7 global features and 10 local features for each fully reconstructed neuron. Each of the local features was represented by four statistical characteristics: minimum, maximum, mean, and standard deviation, resulting in a total of a 47-dimensional feature vector. The seven global features are ‘Stems’, ‘Branches’, ‘OverallWidth’, ‘OverallHeight’, ‘OverallDepth’, ‘OverallVolume’, and ‘Length’, which are defined in L-Measure. The ten local features are ‘br_length’ (path length of a branch), ‘br_order’ (branch order), ‘br_contraction’ (contraction of a branch in L-Measure), ‘bif_EucDist2soma’, ‘bif_PathDist2soma’, ‘asymmetry’ (‘Partition Asymmetry’ in L-Measure), ‘ampl_local’, ‘ampl_remote’, ‘tilt_local’, and ‘tilt_remote’ (Supplementary Figure S6). Next, we standardized all the features using Z-score normalization to ensure that they were in the same scale, and evaluated their similarities as the cosine distances between these features. We also considered the spatial relationships between neurons by calculating the Euclidean distance (d) of each soma in CCFv3 space. We then computed the exponential of -d for these two somas after normalization. We defined the similarity of neurons as the product of feature similarity and spatial distance. Spectral clustering was utilized to classify neurons into different clusters. Specifically, we treated the entire dataset as a graph, where the neurons in the dataset served as individual nodes, and the weights of the connections between nodes were defined by the similarities between the neurons. The silhouette scores between class pairs’ soma locations were estimated with the ‘metrics.silhouette_score’ method of the scikit-learn package, using default parameters, which are calculated as $\left(b-a\right)\;/\;max(a,\;b)$, where (a) is the mean intra-cluster distance of a sample, and (b) is the distance between the sample and the cluster not containing the sample

### Arbor detection and analysis

We adopted a slightly different definition of arbor, referring to a relatively dense-packed sub-tree, rather than the traditional entire dendritic or axonal tree. The apical and basal dendritic arbors were the complete apical and basal dendrites. For axons, we utilized spectral clustering to subdivide them into arbors. This was done by creating an undirected graph composed of vertices that represent nodes in the original tree, while the weights of edges between vertex pairs were represented by the exponential of the negative distances between nodes. Our implementation allowed for the subdivision of the entire neuron into closely packed arbors while preserving inter-node connectivity. To facilitate comparison, we utilized the dominant auto-clustering arbor number of neurons in the same brain region using the majority-vote principle. Regional features were calculated by taking the average of the features of all neurons in that particular region.

We employed the number of branches (‘#branch’), total arbor volume (‘volume’), and maximal spatial density (‘max_density’) - the latter being defined as the number of axonal nodes located within a 20 μm radius for every node - as three features to characterize arbor morphology. We differentiated between proximal and distal arbors based on whether or not the Euclidean distance from the maximal density node to the soma exceeded 750 μm. All values of the same feature were min-max normalized to the range of 0 to 1. The arbors of each neuron were sorted by the Euclidean distance-to-soma and designated as ‘A1’, ‘A2’, and ‘A3’ (if present). To maintain coherence with the CCFv3 convention, we followed the same nomenclature for all regions, and the suffix ‘ET’ and ‘IT’ represented the extratelencephalic and intratelencephalic projection subtypes, respectively. For the classification of thalamic axonal arbors, we utilized agglomerative clustering by combining 4 features (the 3 aforementioned features and distance-to-soma) alongside the average projection of the axonal arbors.

### Detect structural hubs

Based on the assumption that axonal neurites in close proximity are more likely to form functional units, we extracted a new sub-neuronal structure called “hub” (Supplementary Figure S10). A hub is defined as a connected component in 3D image space enclosing neighboring high-density nodes. To accomplish this, we rescaled and resampled the axons into 80 μm spaced representation in the low-resolution (25 μm) coordinate system, and initialized an empty unsigned 8-bit integer image sized to the tight bounding box of all neuronal nodes. Next, we calculated the spatial node density as the number of neuronal nodes within a 500 μm sphere centered at each node. Nodes with a neighboring node count exceeding 70 (density of 134 nodes per mm^3^) were identified as high-density nodes, and their corresponding voxels in the image were set to 255 (foreground value). Subsequently, we applied a 3-dimensional morphological dilation with a 10×10×10 (voxels) kernel, equivalent to a 250×250×250 (μm) square, to merge nearby high-density nodes. Finally, all connected components in the image were identified as hubs.

### Detect primary axonal tract motifs

The primary axonal tract for a neuron is the longest axonal path without short branches at the terminal side. The process of identifying the primary axonal tract begins by determining the longest axonal path and then iteratively removing all branches shorter than the second-longest axonal branch from the tip of the path towards its soma. The resulting path is the primary axonal tract, and its direction is defined as soma to the terminal.

We then mapped primary tracts to the standardized CCFv3 space, grouping them according to projection subtypes, and estimated the radius profile of each group. To this end, we sub-sampled each tract with 200 uniformly-spaced nodes and calculated the cross-sectional radii of points with corresponding percentiles. In specific, for a given set of points, we computed the distance of every point to their center in the reduced 2-dimensional space generated by principal component analysis (PCA), and extracted the 75th percentile as the radius. Based on the comparison of somas and terminal radii, projection patterns were defined as convergent, divergent, and parallel.

### Detect axonal boutons

We updated the approach reported in (Jiang et al., 2022) by combining both intensity and radius profiles along the axonal shafts. The detection process starts with partitioning the axonal skeletons into 20 μm length fragments, along which we calculated the intensity and radius profiles, leading to the identification of initial candidates for axonal boutons, which exhibit overlapped peaks in the intensity and radius profiles. False positive results in the initial candidates were filtered out through heuristic criteria that an axonal bouton should be 1.5 times larger than its surrounding axonal nodes and have an image intensity value above 120 in 8-bit images (maximum intensity 255). Finally, we remove any possible duplicates by deleting candidates that were closer than five voxels. All detected boutons were registered to the CCFv3 atlas along with their morphologies using mBrainAligner.

### Cross-scale feature generation

We obtained a comprehensive 84-dimension cross-scale feature vector (Figure 1E) for each neuron in SEU-A1891, by concatenating features derived from five distinct morphological scales, including microenvironment, full morphology, arbor, bouton, and primary axonal tract (motif). The microenvironment features for each neuron were acquired by extracting the microenvironment features of the neuron in the same soma region in SEU-D15K that had the most similar L-Measure features. For microenvironment and full morphology, we utilized 18 L-Measure features as those in “Dendritic microenvironment construction” (Methods). The arbor features consisted of a concatenation of apical (if present, otherwise zero features were used), basal, and axonal arbor features. For comparative reasons, the axons of each neuron were arborized to two arbors. All features were standardized to a normal distribution and concatenated. Features of a region were estimated by averaging all neurons within that region.

We introduced a new metric, called the DS matrix, to calibrate the diversity among cell types, encompassing both intra-type and inter-type similarities. Each value (DS value) in the matrix indicates the average correlation coefficient between all neuron pairs for the respective two regions (neuron types). The correlation of two neurons is determined by calculating the Pearson correlation coefficient between their cross-scale features. A higher value in the matrix signifies greater stereotypy, while a lower value signifies greater diversity. The axon-axon “distance” was the Euclidean distance between the projection vectors of the neuron pair, which was released in our previous work (Peng et al., 2021).

## Supplementary Material

Supplement 1

## Data Availability

A complete list of the brains and their meta information are summarized in Supplementary Table S1). The morphometric data are deposited on Google Drive (https://drive.google.com/drive/folders/1NwwTe840_0KQhv-zVLhw58LU9nntkb-F?usp=sharing), including all annotated somas (SEU-S227K, Soma_morphometry.xlsx), 1891 full morphologies along with their arborizations, primary axonal tracts, and putative synaptic boutons (SEU-A1891, https://drive.google.com/drive/folders/1sK8sv01cGhHmI3lBnn0tthaT0PeD_6sV?usp=share_link, with their meta information summarized in: Full_morphometry.xlsx), and the automatic traced local morphologies (SEU-D15K, https://drive.google.com/drive/folders/1snCS2AqNon9_UjiiibWLSIUKkOAb1YeE, with its meta information summarized in: Soma_morphometry.xlsx). For most of these data, both their raw versions (morphometry in original image space) and standardized versions (in CCFv3 coordinate system) are provided.
