# Supplementary material for: Full-Spectrum Neuronal Diversity and Stereotypy through Whole Brain Morphometry
Source: Res Sq. 2023 Jul 25:rs.3.rs-3146034. Preprint. [Version 1] doi: 10.21203/rs.3.rs-3146034/v1 (PMC10402258; doi:10.21203/rs.3.rs-3146034/v1)
Supplement: Supplement 1 [file NIHPPrs3146034v1-supplement-1.pdf]

Supplementary Figures

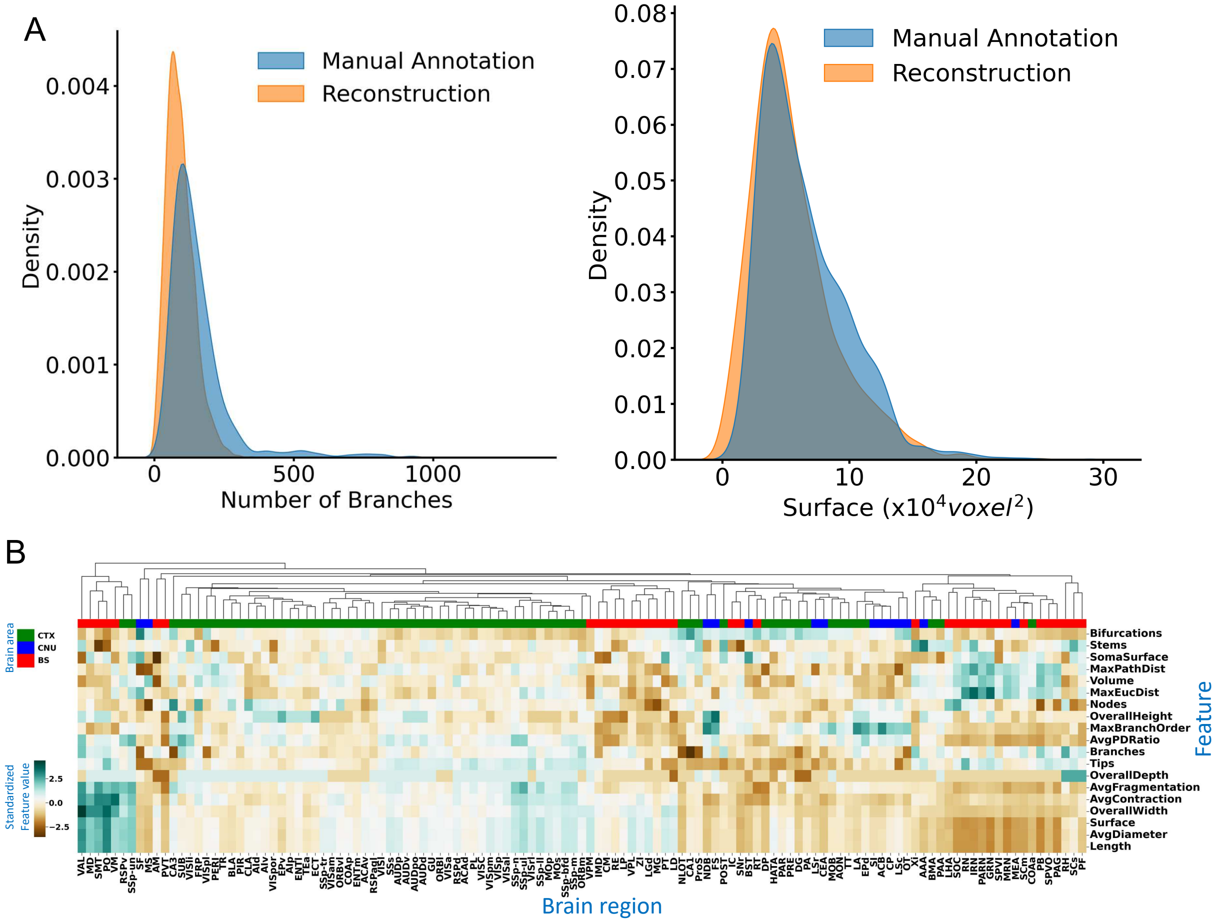

Supplementary Figure S1. **Verification of the auto-traced local morphologies based on L-Measure features. A**. The distributions of two critical morphological features, namely the number of branches and total surface, were compared between the auto-traced reconstructions and manual annotations (gold standards) within image blocks in the shape of 512×512×256 voxels (*xyz* order, approximately 256×256x512 µm). A total number of 1854 successfully reconstructed local morphologies and their corresponding manual reconstructions were used for comparison. **B**. Hierarchical clustering performed for the 121 CCF-R314 regions that contained at least 10 neurons. The regions containing less than 10 neurons (somas) were discarded, and all other neurons in the SEU-D15K were used in calculating the regional features, which involved three brain areas consisting of functionally related region sets defined in CCFv3, namely cerebellum (CB), cerebral nuclei (CNU), and cortex (CTX). The regions and brain areas were estimated based on soma location after registration to the CCFv3 atlas. The corresponding brain area for each region was listed at the top of the heatmap. The regional features were represented by the median features of all neuronal 19-dimensional L-Measure features in the corresponding regions. The values of each feature were Z-score normalized separately. The neuronal features exhibited a notable aggregation according to brain areas in general.

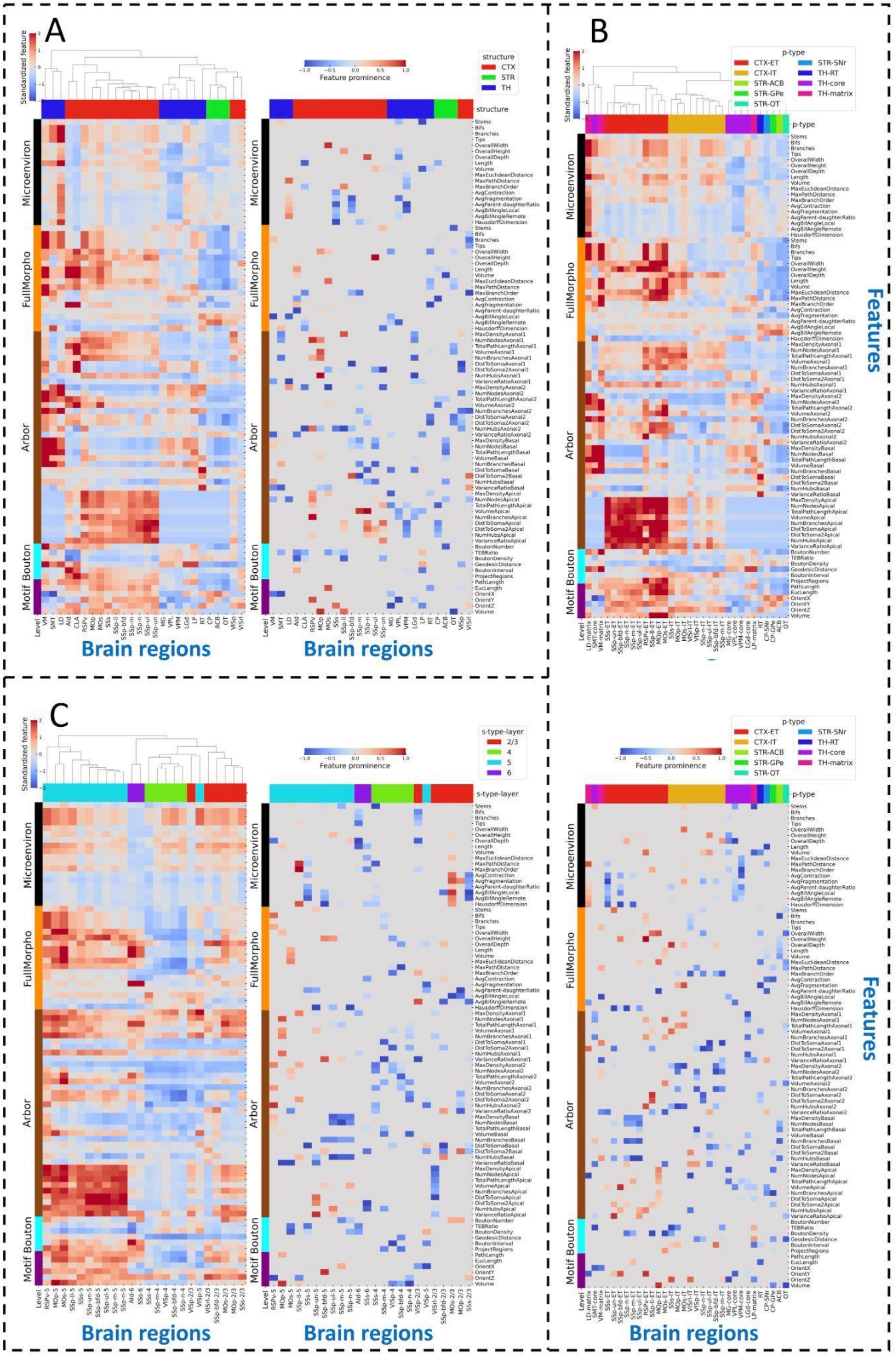
A
B
C
Standardized feature
structure
CTX
STR
TH
Feature prominence
Microenviron
FullMorpho
Arbor
Motif Bouton
Brain regions
Features
p-type
CTX-ET
CTX-IT
STR-ACB
STR-GPe
STR-OT
STR-SNr
TH-RT
TH-core
TH-matrix
s-type-layer
2/3
4
5
6

Supplementary Figure S2. **Cross-scale feature maps of whole-brain neuron types and subtypes. A**. Left: Cross-scale feature map for soma types (s-types) that incorporates five different scales: microenvironment, full morphology, arbor, bouton, and motif. By combining these features, a comprehensive set of cross-scale features is obtained. The values of each feature are Z-score normalized by subtracting their mean value and then dividing by their standard deviation. The right and left y-ticks of the map are the feature names and their corresponding morphometry levels, respectively. Hierarchical clustering is applied to all s-types, and the resulting dendrogram is displayed at the top of the map. The x-ticks are sorted according to the dendrogram. Right: The feature prominence map delineates the ten most discriminating features for each s-type, with the prominence scores determined by the ordering of the absolute feature values, and subsequently max-normalized by dividing 10. The prominence values are colored by the signs of their original features value in the cross-scale feature map, with blue indicating a positive value and red indicating a negative value. **B**-**C** are similar maps for projection subtypes (sp-types) and lamination subtypes (sl-types) of cortical neurons, where ET and IT are the extratelencephalic and intratelencephalic projecting subtypes, and 2/3, 4, 5, 6 are the cortical layers of somas.

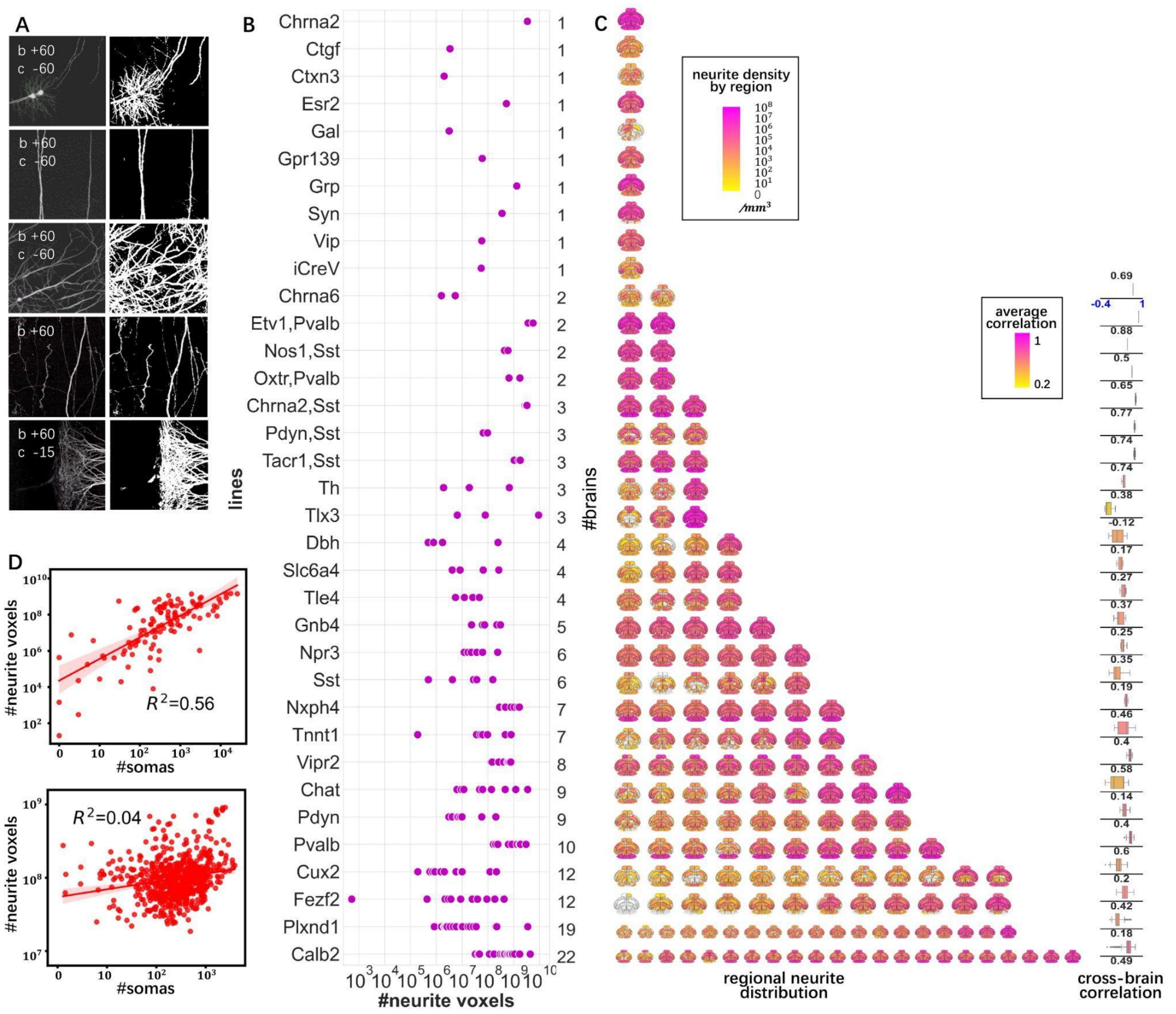

Supplementary Figure S3. **The neurite voxel distributions in fMOST brains. A**. Image blocks exemplifying our neurite segmentation results in fMOST brains. The left and right images are the maximum intensity projections (MIP) of raw images and corresponding neurite segmentation maps. The top left legends on the raw MIPs are the adjustments applied on them for better visualization. 'b' and 'c' represent brightness and contrast adjustment, while '+' and '-' signify percentage changes for the corresponding adjustments. **B**. Distributions of neurite voxel numbers of 177 brains from 35 transgenic mouse lines. The number of neurite voxels is the total number of voxels identified as neurite signals in a brain image. Each magenta dot represents a brain, and the total number of brains in each line is displayed on the right side. **C**. Whole-brain neurite patterns. Zoom-out view of whole-brain neurite voxel distribution at a regional level. Box plots on the right side show the correlations between regional neurite distribution patterns of all brain pairs in each transgenic line. **D.** Top: Relationship between the number of identified neurite voxels and the total number of annotated somas, which exhibits a good linearity with a R_square value 0.56. Bottom: Relationship between the number of identified neurite numbers and the total number of annotated somas for every region.

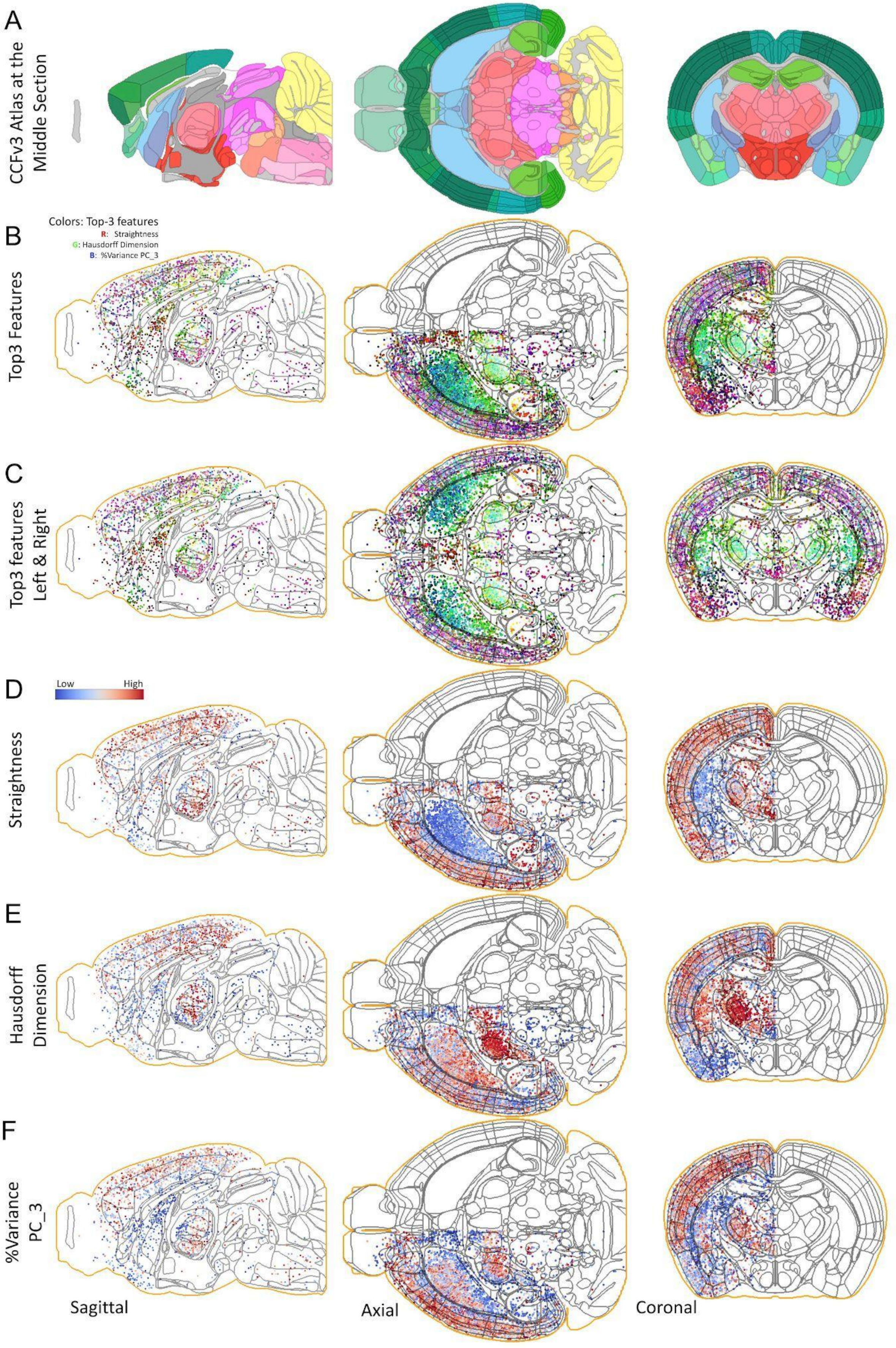
A
CCFv3 Atlas at the Middle Section
B
Colors: Top-3 features
R: Straightness
G: Hausdorff Dimension
B: %Variance PC_3
Top3 Features
C
Top3 features Left & Right
D
Low
High
Straightness
E
Hausdorff Dimension
F
%Variance PC_3
Sagittal
Axial
Coronal

Supplementary Figure S4. **Whole-brain microenvironment feature distributions along middle sections of the sagittal, axial, and coronal views. A.** The sagittal, axial, and coronal middle sections of the CCFv3 atlas. Brain areas and regions are colored following the convention of the CCFv3 atlas. Cortical regions are in green-blue colors, cerebral nuclei regions are in cyan, brain stem regions are in red, midbrain regions are in pink, and cerebellar regions are in yellow. The gray lines are the boundaries of CCFv3 regions. **B**. Projection of the top 3 discriminating morphological microenvironment features selected through minimum Redundancy-Maximum Relevance (mRMR) on the middle sections. The top 3 features are: average straightness, Hausdorff Dimension, and variance percentage of the third component of all nodes, and they are encoded in the red (R), green (G), and blue (B) channels of the image. The feature values are normalized and histogram-equalized to the unsigned 8-bit integer range. Only neurons within a 1-millimeter range in both directions are included. The outermost boundary of the CCFv3 brain template is outlined in orange, and the microenvironments on the right hemisphere are flipped to the left hemisphere. **C**. Similar to panel B, but the right hemispheric microenvironments are not flipped. **D-F**, The distributions for the three features are displayed separately at each view.

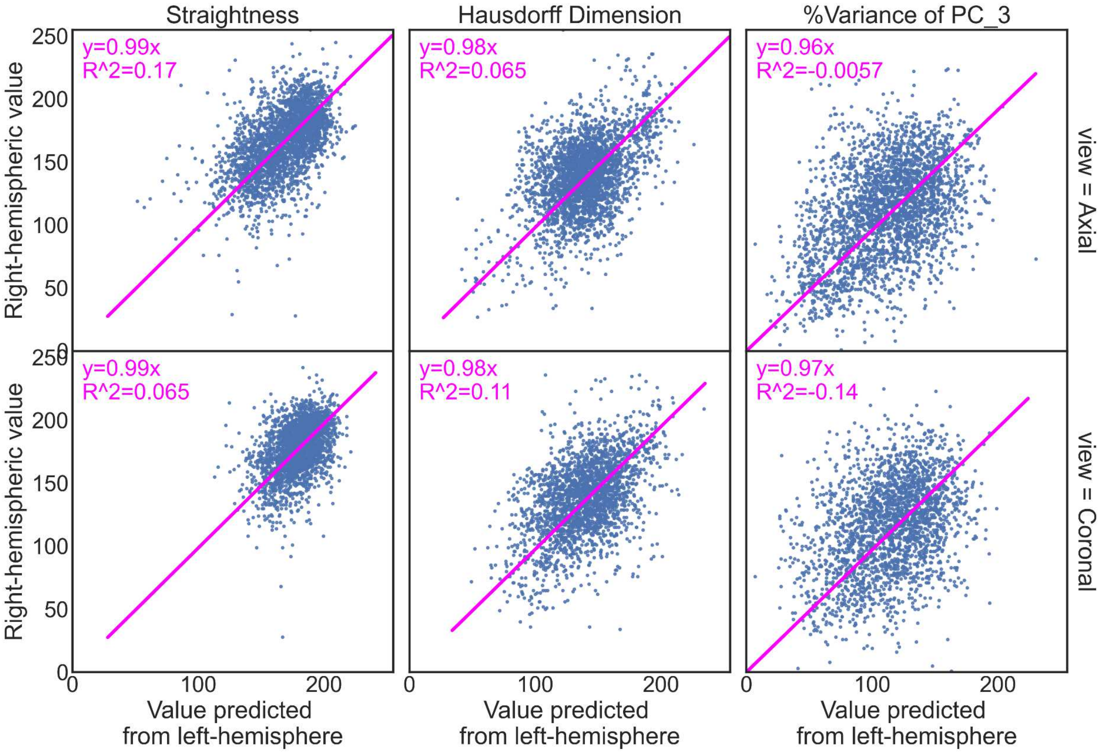

Supplementary Figure S5. **Correlations between feature values in the right hemisphere and those predicted from microenvironments of the left hemisphere on the axial and coronal middle sections.** The values on the y-axis are the feature values of microenvironments in the right hemisphere, while the values on the x-axis are predicted features for mirrored positions through multidimensional linear interpolation using features of the left hemisphere. The points are fitted with the linear function $y = a \cdot x$.

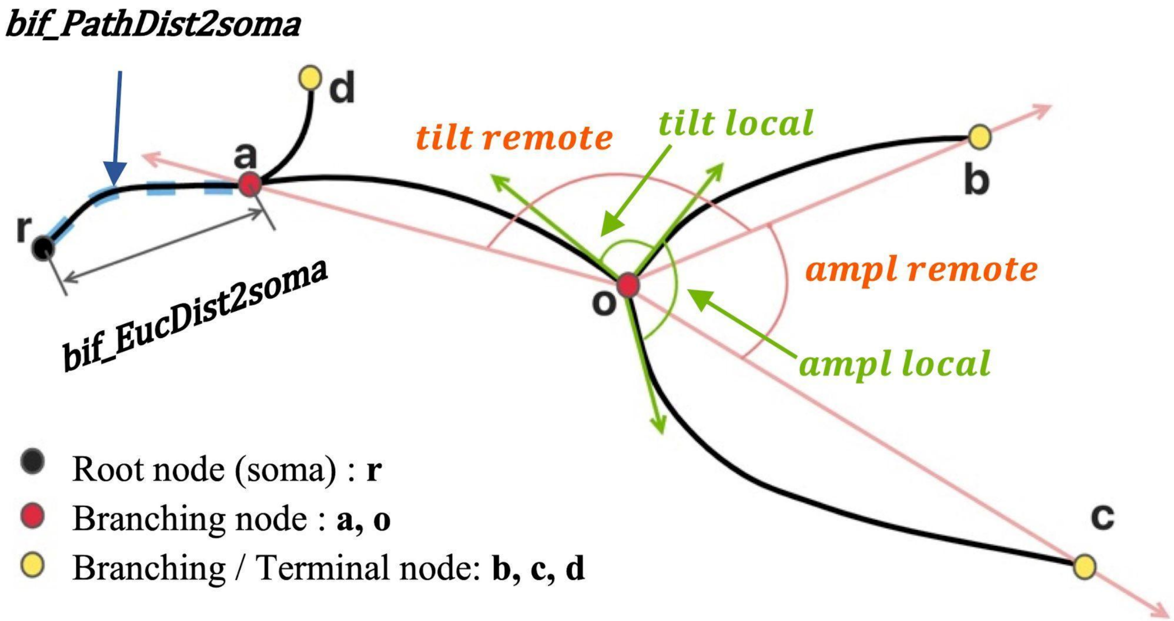

Supplementary Figure S6. **Diagram illustrating the definition of several critical local morphological features leveraged in full morphology analysis.** The features 'bif_EucDist2soma' and 'bif_PathDist2soma' are Euclidean and path distances from the current bifurcation point to the root node (soma). 'tilt remote' is the 'Bif_tilt_remote' defined in L-Measure, which represents the angle between the parent node, the current bifurcation point, and one of its two daughter critical nodes. The smaller angle of the two angles formed with the two daughter nodes is used. A critical node here is a topological critical point that is either a terminating point, a bifurcation point, or a root point. The feature 'tilt local' is similar to 'tilt remote' except the anchor points are not critical points, but instead are the nearest compartments along the branches. The features 'ampl remote' and 'ampl local' are similar to 'tilt remote' and 'tilt local' except that the angle is formed by daughter points and the current branching point.

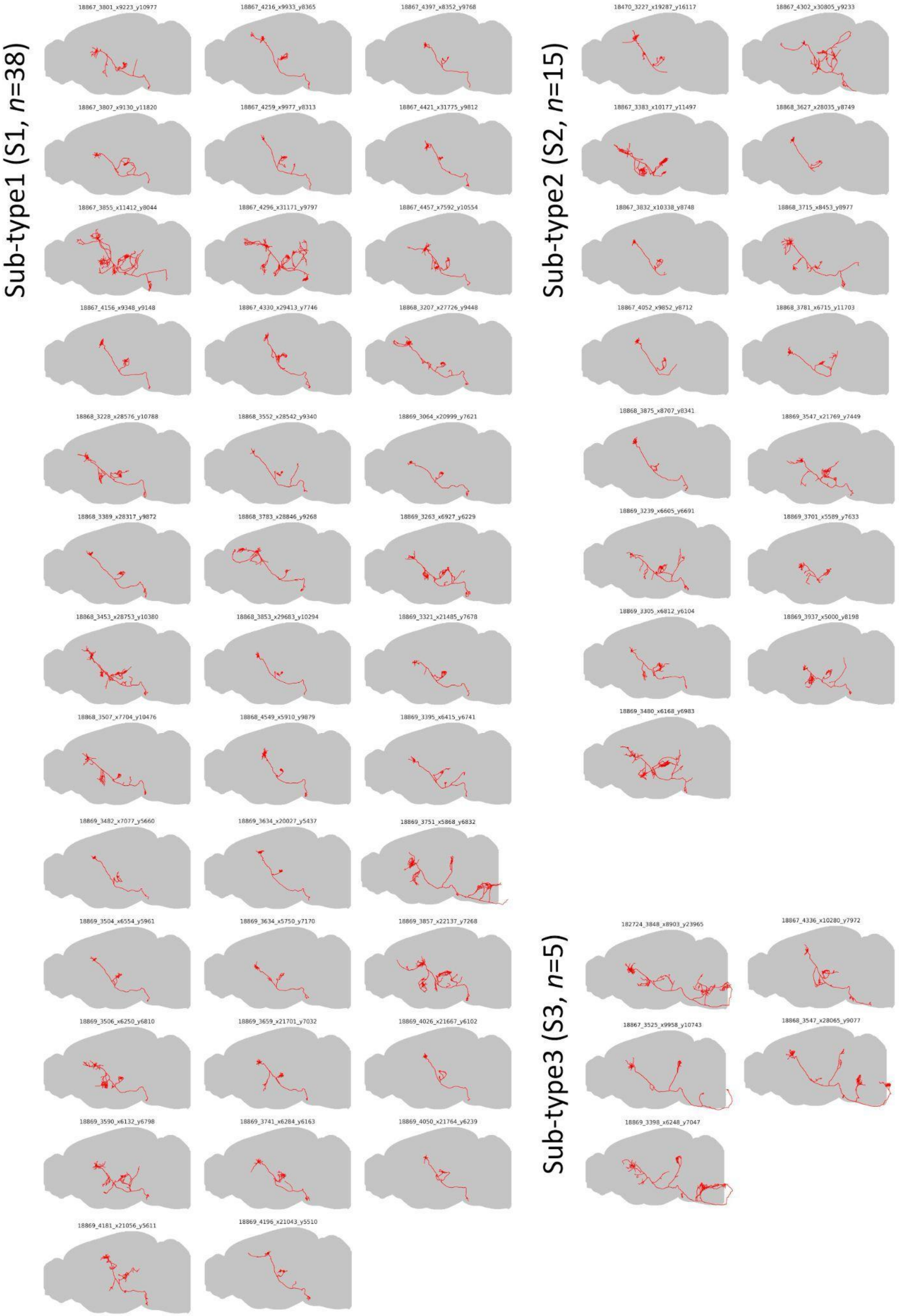
Sub-type1 (S1, n=38)
18867_3801_x9223_y10977
18867_4216_x9933_y8365
18867_4397_x8352_y9768
18867_3807_x9130_y11820
18867_4259_x9977_y8313
18867_4421_x31775_y9812
18867_3855_x11412_y8044
18867_4296_x31171_y9797
18867_4457_x7592_y10554
18867_4156_x9348_y9148
18867_4330_x29413_y7746
18868_3207_x27726_y9448
18868_3228_x28576_y10788
18868_3552_x28542_y9340
18869_3064_x20999_y7621
18868_3389_x28317_y9872
18868_3783_x28846_y9268
18869_3263_x6927_y6229
18868_3453_x28753_y10380
18868_3853_x29683_y10294
18869_3321_x21485_y7678
18868_3507_x7704_y10476
18868_4549_x5910_y9879
18869_3395_x6415_y6741
18869_3482_x7077_y5660
18869_3634_x20027_y5437
18869_3751_x5868_y6832
18869_3504_x6554_y5961
18869_3634_x5750_y7170
18869_3857_x22137_y7268
18869_3506_x6250_y6810
18869_3659_x21701_y7032
18869_4026_x21667_y6102
18869_3590_x6132_y6798
18869_3741_x6284_y6163
18869_4050_x21764_y6239
18869_4181_x21056_y5611
18869_4196_x21043_y5510
Sub-type2 (S2, n=15)
18470_3227_x19287_y16117
18867_4302_x30805_y9233
18867_3383_x10177_y11497
18868_3627_x28035_y8749
18867_3832_x10338_y8748
18868_3715_x8453_y8977
18867_4052_x9852_y8712
18868_3781_x6715_y11703
18868_3875_x8707_y8341
18869_3547_x21769_y7449
18869_3239_x6605_y6691
18869_3701_x5589_y7633
18869_3305_x6812_y6104
18869_3937_x5000_y8198
18869_3480_x6168_y6983
Sub-type3 (S3, n=5)
182724_3848_x8903_y23965
18867_4336_x10280_y7972
18867_3525_x9958_y10743
18868_3547_x28065_y9077
18869_3398_x6248_y7047

***Supplementary Figure S7.* Sagittal projections of the three subtypes of L5 ET-projecting SSp-m neurons in the cortex.** L5 ET-projecting SSp-m neuron is a fine-grain extratelencephalic projecting cortical neuron type SSp-m with the soma located at cortical layer 5 (L5). All 38 subtype-1, 15 subtype-2, and 5 subtype-3 neurons are overlaid on the sagittal view of the CCFv3 template. The three subtypes are classified based on the terminal coordinates of their primary tracts using K-Means clustering.

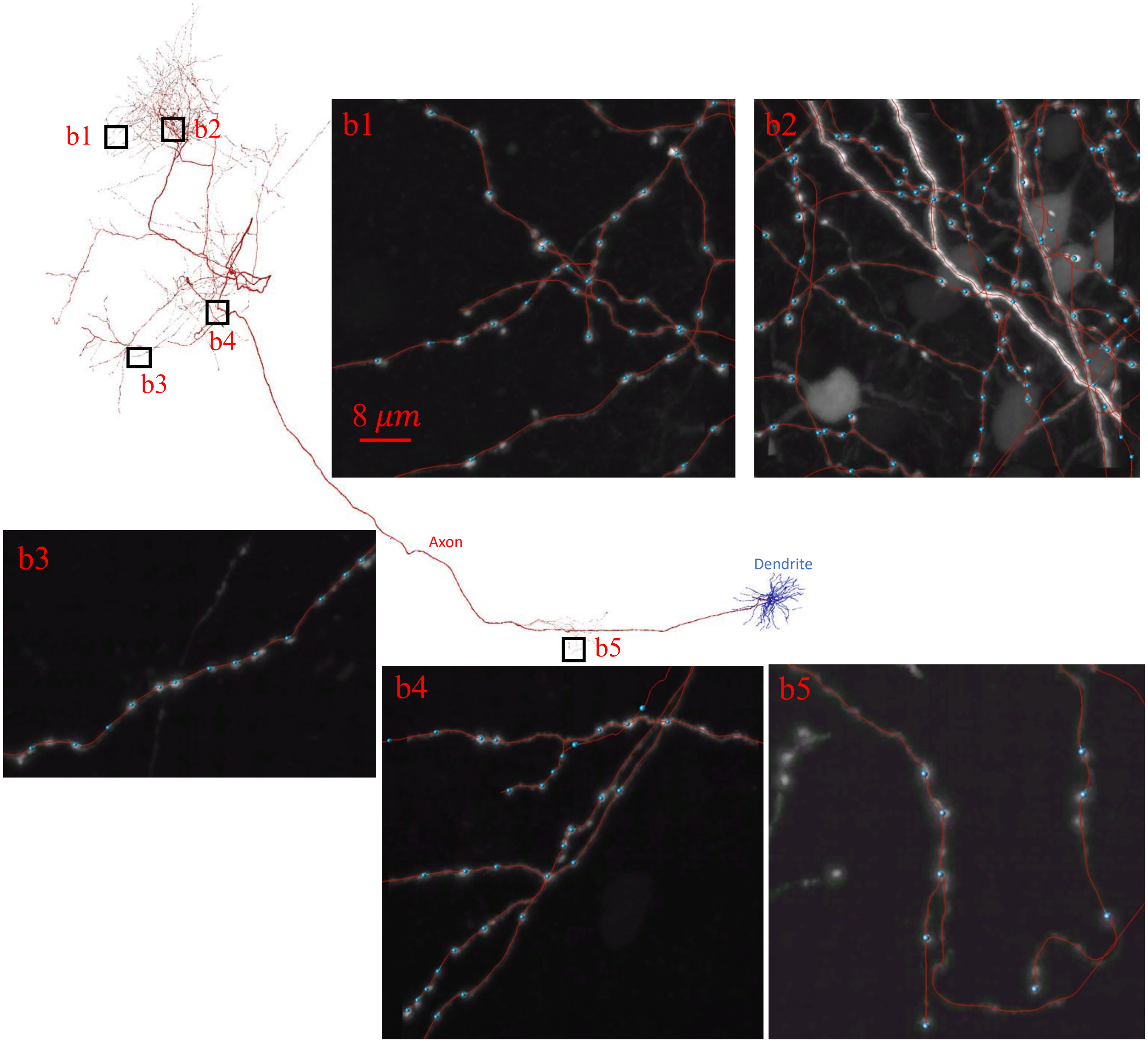

Supplementary Figure S8. **A thalamic VPM neuron with detected boutons overlaid.** Five zoom-in blocks, b1-5, are displayed through maximum intensity projection (MIP), and the reconstructed skeletons are overlaid in place with the image. The cyan dots are the detected boutons. The full morphology of the neuron is illustrated in the middle of these blocks, with dendrites colored in blue and axons in red.

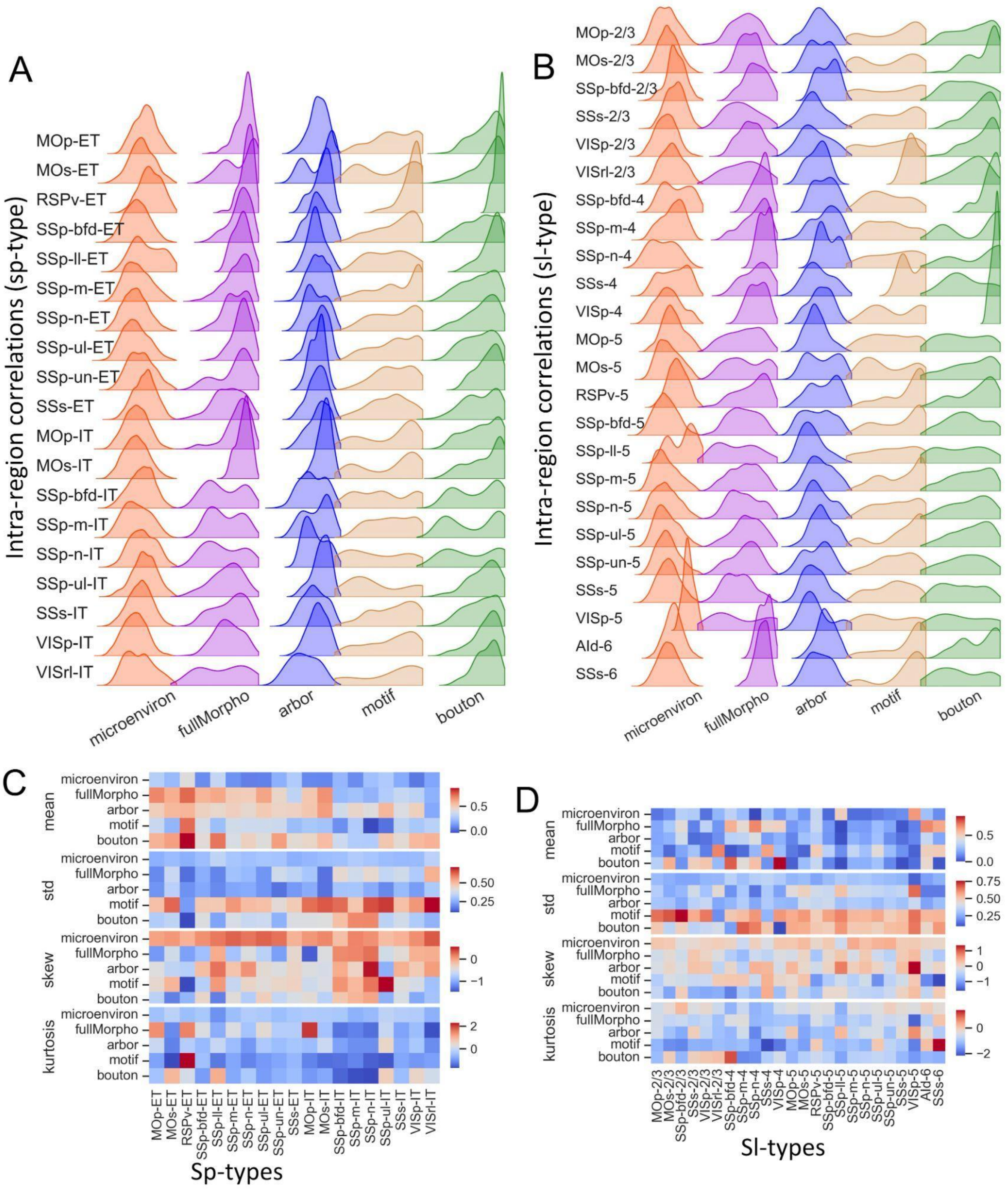

Supplementary Figure S9. Intra-region correlations for projection subtypes (sp-types) and lamination differentiated subtypes (sl-types) of cortical neurons. **A** and **B**. Density plots of the intra-region correlation distributions for sp-types and sl-types at different morphometry levels. **C** and **D** Heatmap of the first (mean), second (std), third (skew), and fourth (kurtosis)-order statistics of intra-regional correlation distributions for sp-types and sl-types respectively.

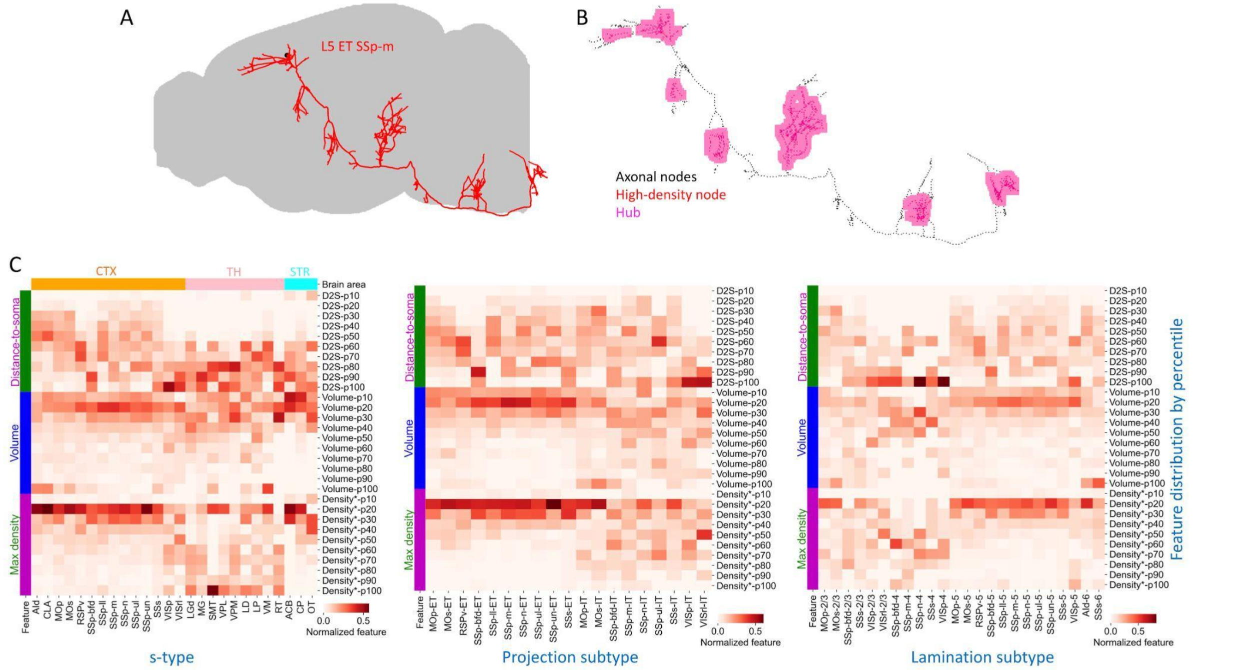

Supplementary Figure S10. **Whole-brain diversity and stereotypy at the sub-neuronal hub level. A**. **Sagittal view of** a L5 ET projection SSp-m neuron overlaid onto the CCFv3 template. **B**. The exemplar neuron comprises 7 hubs, which are sets of densely packed neuronal nodes in spatial proximity. The black dots represent axonal nodes, the red dots represent high-density nodes, and the pink regions indicate detected hubs. A node refers to evenly sampled points on the neuron skeleton, while a high-density node refers to a node that contains a large number of nodes in its spatial vicinity (see **Methods**). **C**. Heatmaps of 3 hub features for soma-types (s-types), projection subtypes, and lamination subtypes. Values for each feature were split into 10 equally sized bins ranging from percentile 10 (P10) to percentile 100 (P100). The three features calculated are 1) Distance to the soma; 2) Volume of the hub in terms of the number of voxels; 3) Maximal node density within a 500 μm range.

Supplementary Table S1 SEU-ALLEN_brains_0615.xlsx
A comprehensive list of the 205 brains used in the study, comprising 191 fMOST brains, 10 STPT brains, and 4 LSFM brains, along with their respective meta information.

Supplementary Table S2 soma_region314_counts.xlsx
Summarization of the total number of somas annotated for all 314 CCFv3 regions (CCF-R314).

Supplementary Table S3 Soma_morphometry.xlsx
A complete list of all 227,581 somas annotated, along with their meta information, including their locations in both the original image space and CCFv3 atlas space, and the corresponding CCFv3 regions based on image registration.

region sets table

Cross-CA

AAA	ACB	MA	SI	ICB	CLA	CP	ENTl	PERI	PYR	FN	FS	GPe	GU	VeCB
MA	FS<br>SI	AAA	ACB<br>FS	VeCB	GU	GPe	PERI	ECT<br>ENTl	UVU<br>FOTU<br>DEC<br>COPY<br>AN<br>PRM<br>FN<br>SIM	PYR	ACB<br>SI	GPi<br>CP	CLA<br>AIp	ICB

— Target region

Intra-CA

ACAd	MT	AHN	AId	AIp	AIv	NR	LIN	APN	RPO	SCm	SCs	SSp-m	SSp-n	SSp-ul
MOs	SNc	MPN	AIv	GU	AId	XII	GRN	PPT	CS	PPT<br>SCs	SCm	SSp-n	SSp-m	SSp-un

SNc	SNr	CA1	VISp	VISal	VISl	VISrl	SPVC	SPVI	SUB	MPN	VISpm	TR	COAp	NTS	CS	OP
SNr<br>MT	SNc	ProS	VISl<br>VISpm<br>VISrl	AUDpo	VISp<br>AUDpo<br>VISli	VISp<br>VISa	SPVI	SPVC	ProS	AHN	VISp	COAp	TR	DMX	RPO	PPT

VPL	VPM	XII	DMX	PAR	RSPd	RSPv	RSPagl	ECT	CENT	ENTm	CUL	DEC	FOTU	UVU	NOD	MOp
VPM	VPL	NR	NTS	ENTm	RSPagl<br>RSPv	RSPd	RSPd	PERI	CUL<br>SIM	PAR	SIM<br>CENT<br>DEC<br>PRM<br>AN	PYR<br>FOTU<br>UVU<br>AN<br>CUL<br>SIM<br>PRM	PYR<br>DEC<br>UVU<br>AN<br>PRM<br>COPY	PYR<br>COPY<br>DEC<br>FOTU<br>PRM<br>NOD<br>AN<br>SIM	COPY<br>UVU	MOs

MOs	SIM	AN	PRM	AUDpo	GPi	COPY	PFL	GRN	FL	PPT
ACAd<br>MOp	CUL<br>AN<br>PRM<br>DEC<br>CENT<br>COPY<br>UVU<br>PYR	PRM<br>SIM<br>COPY<br>PFL<br>DEC<br>FOTU<br>PYR<br>UVU<br>CUL	AN<br>COPY<br>SIM<br>PFL<br>FOTU<br>UVU<br>DEC<br>CUL<br>PYR	VISl<br>VISal	GPe	PRM<br>PFL<br>AN<br>UVU<br>PYR<br>FOTU<br>NOD<br>SIM	COPY<br>PRM<br>AN<br>FL	LIN	PFL	OP<br>SCm<br>APN

SSp-un	VISa	VISli	ProS
SSp-ul	VISrl	VISl	SUB<br>CA1

***Supplementary Table S4.* Highly correlated regions for each target region.** The regions were identified through pairwise correlation of cross-brain regional neurite density patterns, which were represented by a 191-dimension vector. A threshold of 0.8 was used to identify highly correlated region pairs. Each column of the table contains a target region (indicated by a background box colored as its belonging compound area as shown in **Figure 2**) and its highly correlated regions in descending order of coefficients. The table displays the target region and its highly correlated regions in descending order of coefficients, with the top component comprising cross-compound area (cross-CA) region sets, and the intra-compound area (inter-CA) region sets listed in the bottom component. The layout was chosen to match panel B of **Figure 2**.

Initial modules

M1	M2	M3	M4	M5	M6	M7	M8	M9	M10	M11	M12	M13	M14	M15	M16
DCO	FN	y	Pa4	TRN	MRN	XII	NLL	LC	IC	OV	SGN	NOT	PVa	SO	LHA
IP	COPY	ICB	PAG	PRNr	RN	MDRNd	SOC	MEV	I5	AVPV	MG	LGd	PD	AVP	LPO
DN	PFL	SUV	DR	PRNc	III	GRN	NTB	SG	PARN	VLPO	AOB	SCs	ARH	MPO	BST
	UVU	VeCB	RPO	Acs5	MA3	IRN	SUT	PCG	SPVC	SCH	MOB	MPT	ASO	MEPO	PeF
	AN	VCO	CS	PC5	RL	MDRNv	CUN	PDTg	SPVI	PVpo	MEA	OP	PT	NDB	LSv
	PRM	LAV	B	V	EW	NR		IF	DMX	VMPO	BA	PPT	PVT	ADP	MS
	DEC	SPIV	SLD	P5		RO		CLI	PGRNd		LH	IntG	VMH	PS	IMD
	FOTU	ECU	AT	RM		AMB		RPA	ISN		MH	IGL	Xi		PVHd
	PYR	MV	VTN	VI		LIN		SLC	PAS			LGv	PVi		BAC
	CENT	x	LDT	PPY		VII		ACVII	Pa5			SubG	SBPV		SCO
	CUL		NI			MARN		DT	PB			LP	RCH		TU
	SIM		DTN			PGRNI		IPN	AP			APN	MPN		TMv
	LING					IO		PN	GR			SCm	DMH		PMv
	NOD					LRN			PSV				PVH		PVp
	FL								SPVO				AHN		TMd
									PRP						
									NTS						
									CU						

M17	M18	M19	M20	M21	M22	M23	M24	M25	M26	M27	M28	M29	M30	M31
CEA	PST	PA	SSp-tr	VISp	ACAd	ENTl	SUB	SSp-m	SAG	VPM	PG	RE	LM	PAA
IA	MT	TR	VISam	VISpm	ACAv	ECT	HATA	SSp-n	PBG	PO	AD	AM	MM	BMA
AAA	RR	COAp	VISrl	VISli	FRP	PERI	DG	SFO	IG	ZI	AV	IAD	SUM	NLOT
MA	PPN	RSPd	VISa	AUDp	PL	PAR	APr	ME	ILA	RT	CL	MD	PH	COAa
ACB	PSTN	RSPv	SSp-ll	AUDd	LD	ENTm	PRE	GPe	DP	VPL	VPMpc	CM	PMd	LSc
OT	VTA	CA3	SSs	VISal	ORBm	TEa	CA1	GPi	MOp		PF	PR	NPC	SF
SI	SH	POST	SSp-bfd	AUDpo	ORBvl	AUDv	ProS	STN	MOs		VPLpc	POL	SPFm	TRS
FS	SPFp		SSp-ul			VISpl		SNc	ORBl		PCN	PoT	SPA	LA
LSr	NB		SSp-un			VISpor		SNr	PIR		RH	LT	IV	BLA
AON						VISl		CP	GU		SMT	PP	IAM	EPv
TT						RSPagl		CLA	AId		VAL	PIL		
								EPd	AIv		VM			
								CA2	AIp					
								FC	VISC					

Tight modules

Cross-CA

M2*	M3*	M17*	M19*	M23*	M25*
FN	ICB	AAA	TR	ENTl	SSp-m
COPY	VeCB	MA	COAp	ECT	SSp-n
PFL		ACB	RSPd	PERI	GPe
UVU		SI	RSPv	PAR	GPi
AN		FS		ENTm	SNc
PRM					SNr
DEC					CP
FOTU					
PYR					
CENT					
CUL					
SIM					
NOD					
FL					

Intra-CA

M4*	M7*	M10*	M13*	M14*	M20*	M21*	M24*	M26*	M27*
RPO	XII	SPVC	SCs	MPN	VISrl	VISp	SUB	MOp	VPM
CS	GRN	SPVI	OP	AHN	VISa	VISpm	CA1	MOs	VPL
	NR	DMX	PPT		SSp-ul	VISal	ProS	GU	
	LIN	NTS	APN		SSp-un	AUDpo		AId	
			SCm					AIv	
								AIp	

Supplementary Table S5. **Compositions of initial and tight modules.** The top cell of the table lists the initial modules, with each column representing a separate module. In the bottom cell, tight modules are listed with the first row comprising cross-CA modules, which involve brain regions from different compound areas. The second row comprises intra-CA modules, which involve brain regions within the same compound area. The module names and orderings are determined by their hierarchical levels in the dendrogram shown in **Figure 2**.

Supplementary Files

This is a list of supplementary files associated with this preprint. Click to download.

- TableS1SEUALLENbrains0615.xlsx
- TableS2somaregion314counts.xlsx
- Tables3Somamorphometry.xlsx
